# Comorbidities in the diseasome are more apparent than real: What Bayesian filtering reveals about the comorbidities of depression

**DOI:** 10.1371/journal.pcbi.1005487

**Published:** 2017-06-23

**Authors:** Peter Marx, Peter Antal, Bence Bolgar, Gyorgy Bagdy, Bill Deakin, Gabriella Juhasz

**Affiliations:** 1 MTA-SE Neuropsychopharmacology and Neurochemistry Research Group, Hungarian Academy of Sciences, Semmelweis University, Budapest, Hungary; 2 Department of Measurement and Information Systems, Budapest University of Technology and Economics, Budapest, Hungary; 3 Department of Pharmacodynamics, Faculty of Pharmacy, Semmelweis University, Budapest, Hungary; 4 NAP-A-SE New Antidepressant Target Research Group, Hungarian Brain Research Program, Semmelweis University, Budapest, Hungary; 5 Neuroscience and Psychiatry Unit, School of Biological Sciences, Faculty of Biology Medicine and Health, The University of Manchester, Manchester, UK and Greater Manchester Mental Health NHS Foundation Trust, Manchester, UK; 6 MTA-SE-NAP B Genetic Brain Imaging Migraine Research Group, Hungarian Academy of Sciences, Semmelweis University, Budapest, Hungary; University of California San Diego, UNITED STATES

## Abstract

Comorbidity patterns have become a major source of information to explore shared mechanisms of pathogenesis between disorders. In hypothesis-free exploration of comorbid conditions, disease-disease networks are usually identified by pairwise methods. However, interpretation of the results is hindered by several confounders. In particular a very large number of pairwise associations can arise indirectly through other comorbidity associations and they increase exponentially with the increasing breadth of the investigated diseases. To investigate and filter this effect, we computed and compared pairwise approaches with a systems-based method, which constructs a sparse Bayesian direct multimorbidity map (BDMM) by systematically eliminating disease-mediated comorbidity relations. Additionally, focusing on depression-related parts of the BDMM, we evaluated correspondence with results from logistic regression, text-mining and molecular-level measures for comorbidities such as genetic overlap and the interactome-based association score. We used a subset of the UK Biobank Resource, a cross-sectional dataset including 247 diseases and 117,392 participants who filled out a detailed questionnaire about mental health. The sparse comorbidity map confirmed that depressed patients frequently suffer from both psychiatric and somatic comorbid disorders. Notably, anxiety and obesity show strong and direct relationships with depression. The BDMM identified further directly co-morbid somatic disorders, e.g. irritable bowel syndrome, fibromyalgia, or migraine. Using the subnetwork of depression and metabolic disorders for functional analysis, the interactome-based system-level score showed the best agreement with the sparse disease network. This indicates that these epidemiologically strong disease-disease relations have improved correspondence with expected molecular-level mechanisms. The substantially fewer number of comorbidity relations in the BDMM compared to pairwise methods implies that biologically meaningful comorbid relations may be less frequent than earlier pairwise methods suggested. The computed interactive comprehensive multimorbidity views over the diseasome are available on the web at Co=MorNet: bioinformatics.mit.bme.hu/UKBNetworks.

## Author summary

Depression is one of the most common of psychiatric disorders and its causation is correspondingly multifactorial, complex and heterogeneous. It occurs in combination with a number of physical illnesses far more commonly than expected by chance. Such comorbidities may be important clues pointing to shared environmental and genetic risk factors and could identify different causal types of depression. However, a method is still needed to weed out statistically significant pairings that nevertheless arise through indirect routes involving comorbidities between other diseases. We examined the pairwise associations among 247 diseases of 117,392 participants recorded in the UK Biobank database. We found that the great majority of disease associations were indirect consequences of a sparse network of ‘direct’ comorbidities (‘sparse diseaseome’) constructed using probabilistic graphical models (PGMs) within the Bayesian statistical framework. In a depression-related subset of illnesses, we found that several pairwise associations of depression were indirect and due to their comorbidities with obesity which had a strong direct connection with depression. Furthermore, the direct comorbidities in a depression-related subset of disorders, but not the pairwise associations, strongly mapped onto an underlying molecular network (‘interactome’) suggesting that this approach significantly improved correspondence with molecular reality.

## Introduction

It has long been recognised that medical disorders frequently co-occur in the same individual [1] but the significance of comorbidity in revealing shared mechanisms of pathogenesis and outcome is a more recent realisation [2–4]. For a given disease or for a focused disease group, the exploration of comorbidities is largely hypothesis driven, together with the cautious selection and management of potential confounders [4–7]. The availablility of large health data sets with full multimorbidity information provides an unprecedented opportunity to understand the overall network of dependencies underpinning complex multimorbidities. These multivariate dependencies in turn become new targets for drug development and other therapies for multimorbid conditions, particularly relevant in aging societies [8–10]. However, the dissection of comorbidity relations is hindered by myriads of confounding factors [11–13]. Following the characterization from Bagley et al. [12], epidemiological co-occurrences can arise through different routes: 1) shared genetic background, 2) disease interactions (a disorder directly causes another), 3) common environmental cause and 4) different biases (diagnosis artifacts, selection biases). Earlier diseasome-wide works focused on the exploration of shared genetic background (1) behind comorbidities [2, 3, 12, 13] and the underlying molecular networks [3, 14–18]. These works relied on pairwise comorbid relations partly controlled for potential confounding factors such as age (for controlling with disease onset see e.g. [2], for incidence-based control see e.g. [12]). However, these approaches do not address the issue of apparent comorbidity mediated by intervening associations with other diseases; a problem of indirect relations that is already attracting attention in other areas of network science [19].

In this paper we demonstrate that probabilistic graphical models (PGMs) in the Bayesian statistical framework provide a principled, unified solution for filtering such disease-mediated indirect relations, for correcting for potential external confounders and for coping with limitations and uncertainty of the data. Specifically, we construct sparse multimorbidity maps by applying PGMs for all diseases, i.e. for the whole diseasome. To our knowledge, this method has not been applied for the diseasome so far, despite the unique ability of PGMs to represent maximally sparse models, demonstrated on, for example genomic datasets [19–25]. Our diseasome-wide evaluations show that this approach efficiently scores and discriminates direct and disease-mediated indirect comorbidity relations and has resulted in a loss of more than 80% of comorbidity relations from prevailing pairwise methods.

We made a more detailed investigation of BDMMs in the subset of psychiatric and metabolic disorders of the diseaseome. We focused on depression, which is a common psychiatric disorder with a complex neurobiological and psychosocial background [26, 27], with approximately 10% prevalence worldwide, and according to forecasts depression will be the largest contributor to the disease burden in the middle- and high-income countries by 2030 according to the World Health Organization [28–30]. Many epidemiologic studies have reported high comorbidity between mental illnesses [5, 31], which was partly explained by shared heritability between psychiatric disorders [32, 33]. Less is known about the complex biopsychosocial mechanisms which underlie associations between somatic and common psychiatric disorders: depression frequently co-occurs with a wide range of somatic disorders, for example with migraine [34], with other disorders causing chronic pain [35, 36], and with cardio-metabolic syndromes [37]. It has been also demonstrated that patients with depression have increased number of diagnosed disorders compared to non-depressed patients [5, 38] and depression worsens the treatment outcome of the comorbid conditions [39] and is an independent predictor of increased mortality rate [40, 41]. Therefore besides the exploration of further comorbidities of depression, it is equally important to discriminate its comorbidities as direct and indirect comorbidities. Such discrimination could reveal more specific pathophysiological subgroups of this heterogeneous condition and thus transform the power of genetic and epidemiological studies to advance precision medicine in psychiatry and metabolic disorders.

We also evaluated the correspondence of BDMMs with molecular-level measures and relations, such as genetic overlap and the interactome-based association score for a depression-related subset of diseases. Focusing on depression we identified a direct multimorbid neighbourhood and confirmed that direct comorbidities correspond to direct relationships in the molecular interactome.

## Results

**Fig 1:**
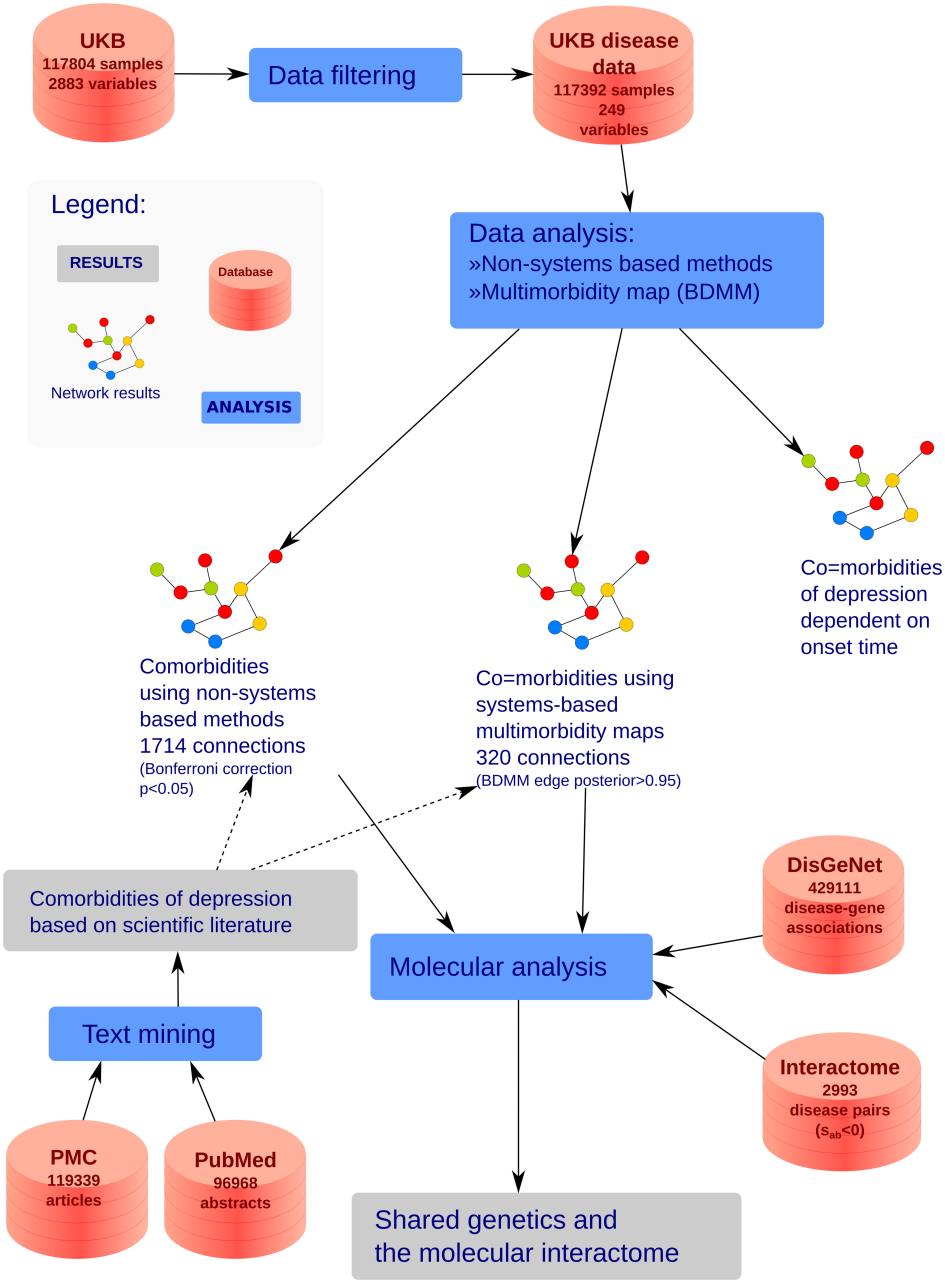
Workflow of the evaluation. The analysed databases and applied methodologies. Solid lines show the route of the data whereas dotted lines represent expert validation.

We used a subsample of 117,392 subjects from the large-scale cohort of the UK Biobank resource (http://www.ukbiobank.ac.uk/) in which the presence or absence of lifetime depression had been established. In the analysis we used 247 diseases with sex and age information, for the construction of this dataset *D*_*UKB*_, see Materials and methods. At the diseasome level, we computed and cross-compared pairwise comorbidity measures and measures of direct and indirect comorbidity relations using a Bayesian systems-based approach. Focusing on depression, we calculated co-occurence based measures for comorbidities of depression using the literature and also logistic regression based measures for depression comorbidities using the UKB data set *D*_*UKB*_. For a depression related subset of diseases, we also evaluated the correspondence of the disease-disease relations with molecular-level measures, including genetic overlap and the interactome-based association score. Fig 1 shows the outline of the applied approaches and their main parameters. In the paper we use interchangeably disease, disorder and morbidity.

**Fig 2:**
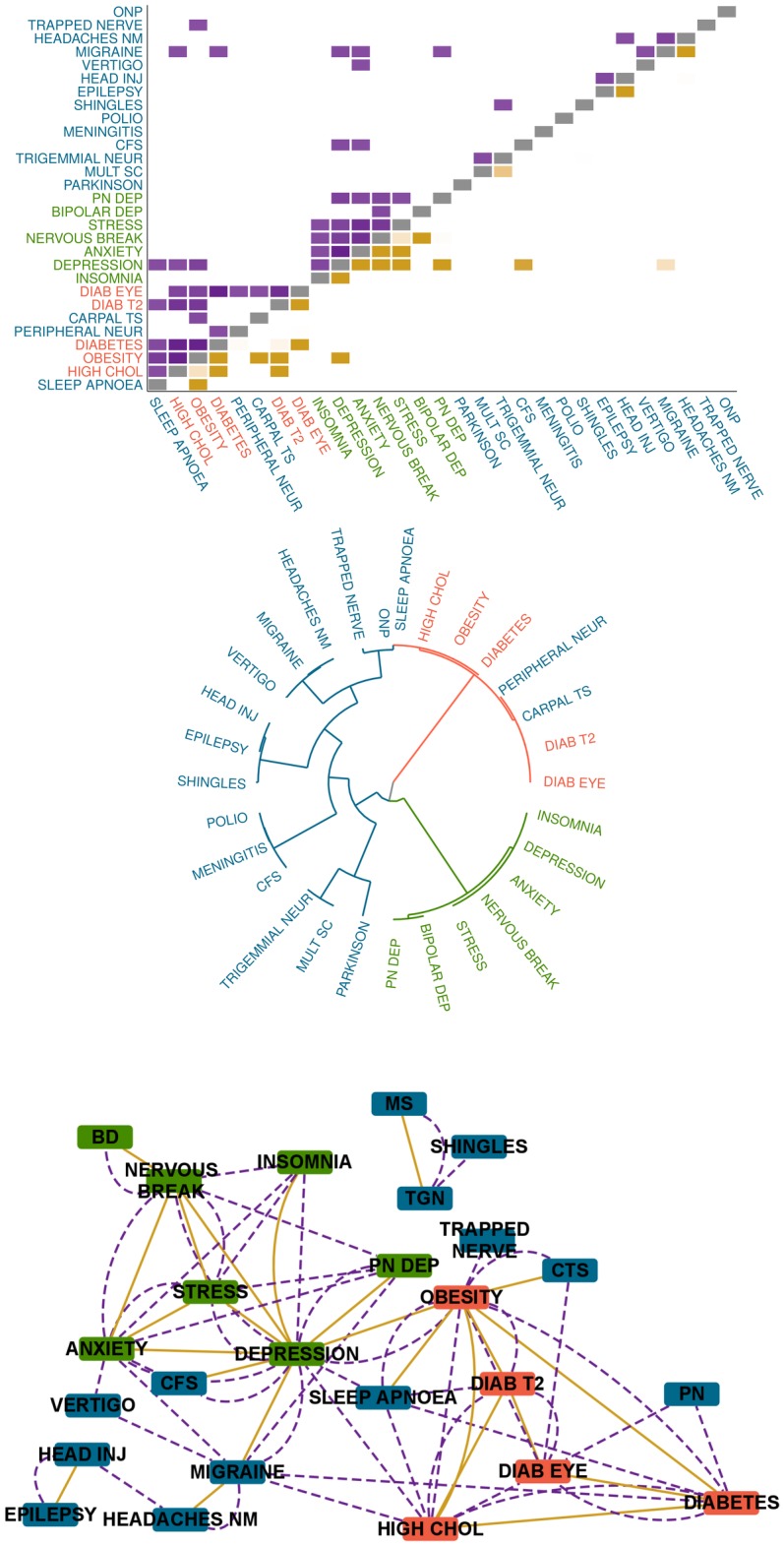
A matrix, tree and network view of comorbid relations. Sparsity and correspondence of pairwise associative measure of comorbidity and co=morbidity posteriors of the Bayesian direct multimorbidity maps (BDMM) using three subsets (clusters) of disorders, namely metabolic syndromes (red), diseases of the nervous system (blue) and mental and behavioural disorders (green), reported in the UK Biobank dataset. Top figure a. shows the p-values of the comorbidity associations by *χ*^2^ test in purple as pairwise statistical associations, while the posterior probabilities of co=morbidities derived from the BDMM are in gold below the gray diagonal. Middle figure b. as intermediate step towards structural dependencies represents the hierarchical clustering of diseases based on the pairwise associations (*χ*^2^ p-values as distances are used by the Ward method to compute a hierarchical clustering) resulting three main clusters, which follows the expected disease groups. Bottom figure c. represents the disease networks, where the gold edges show the sparse co=morbidities in BDMM while the purple dashed lines show indirect links defined by pairwise methods. We used the following abbreviations for the disease names: ANXIETY: anxiety/panic attacks, CTS: carpal tunnel syndrome, CFS: chronic fatigue syndrome, DIAB EYE: diabetic eye disease, HEADACHES NM: headaches (not migraine), HEAD INJ: head injury, HIGH CHOL: high cholesterol, BD: mania/bipolar disorder/manic depression, MS: multiple sclerosis, NERVOUS BREAK: nervous breakdown, ONP: other neurological problem, PD: Parkinson’s disease, PN: peripheral neuropathy, POLIO: polio/poliomyelitis, PN DEP: post-natal depression, TGN: trigeminal neuralgia.

To explore direct and indirect comorbidities, we used Bayesian networks (BN) in the Bayesian model averaging framework [20, 21, 23–25, 42, 43]. In the resulting BDMM an undirected edge denotes a pairwise ‘co=morbidity’ relation, which corresponds to the presence of an edge in any orientation between the respective morbidities in the underlying BN. Thus a co=morbidity relation represents direct, unmediated dependence between two disorders, which cannot be blocked by other diseases (note that this undirected skeleton of a BN does not have the d-separation based semantics and interpretability as dependency or independency map [43]). An indirect comorbidity denotes a relationship without direct connection where one or more diseases directly connect or confound the two disorders (see S1 Appendix) [13]. To quantitatively characterize the plausibility of direct and indirect comorbidity relations in this systems-based approach, we used their respective a posteriori probabilities from Bayesian model averaging. Fig 2 illustrates differences between pairwise and systems-based approaches to explore multimorbidities on a restricted subset of diseases.

Finally, we also performed multiple analysis with depression as a target: we used text-mining of relevant PubMed abstracts and PMC articles to summarize the known comorbid disorders of depression based on co-occurrence measures, which were compared to data-based measures, and we also investigated the effect of onset time of depression on the BDMMs approach.

### Comorbidities in the diseasome: Direct or indirect?

**Fig 3:**
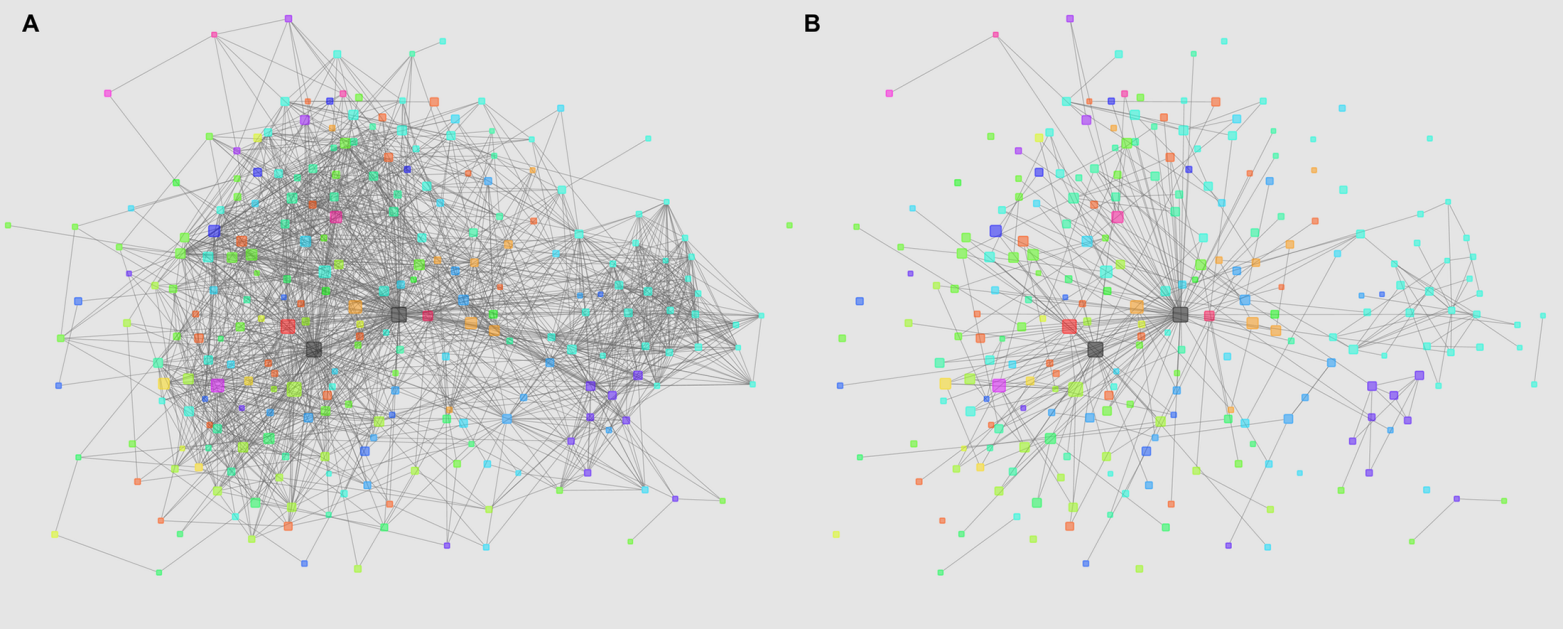
Sparsity of Bayesian direct morbidity maps of multimorbidities over the diseasome. Figure A shows the network of disorders based on *χ*^2^ independence tests whereas figure B represents the sparser BDMM of the same disorders. The node color denotes the different high level ICD-10 categories of the different disorders. The node size is proportional to the prevalence of the diseases. The two gray nodes with multiple connections are sex and age.

To explore the direct and indirect status of comorbidities in the diseasome and to investigate the effect of filtering disease-mediated, indirect comorbidity relations by BDMMs, we computed multiple pairwise measures. The prevailing non-systems based approaches usually use a pairwise measure such as odds ratio (OR), Pearson’s correlation coefficient (Φ) or logistic regression to determine the epidemiologic relationship of disorders [2, 3]. We computed these most often used statistical descriptors of comorbidity for all the investigated diseases (see S2 Dataset for all computed pairwise measures for all possible disease pairs). We also computed BDMMs and cross-compared with the prevailing pairwise approaches [3, 11, 12, 18]. The Fig 3 illustrates the sparsity of the direct (systems-based) map compared to a non-systems based network over the diseasome. The BDMM approach resulted in 320 direct connections (BDMM edge posterior > 0.95) whereas applying the *χ*^2^ independence test 1714 disorder-disorder relations have significant p-values with a threshold of 0.05 after Bonferroni correction.

**Fig 4:**
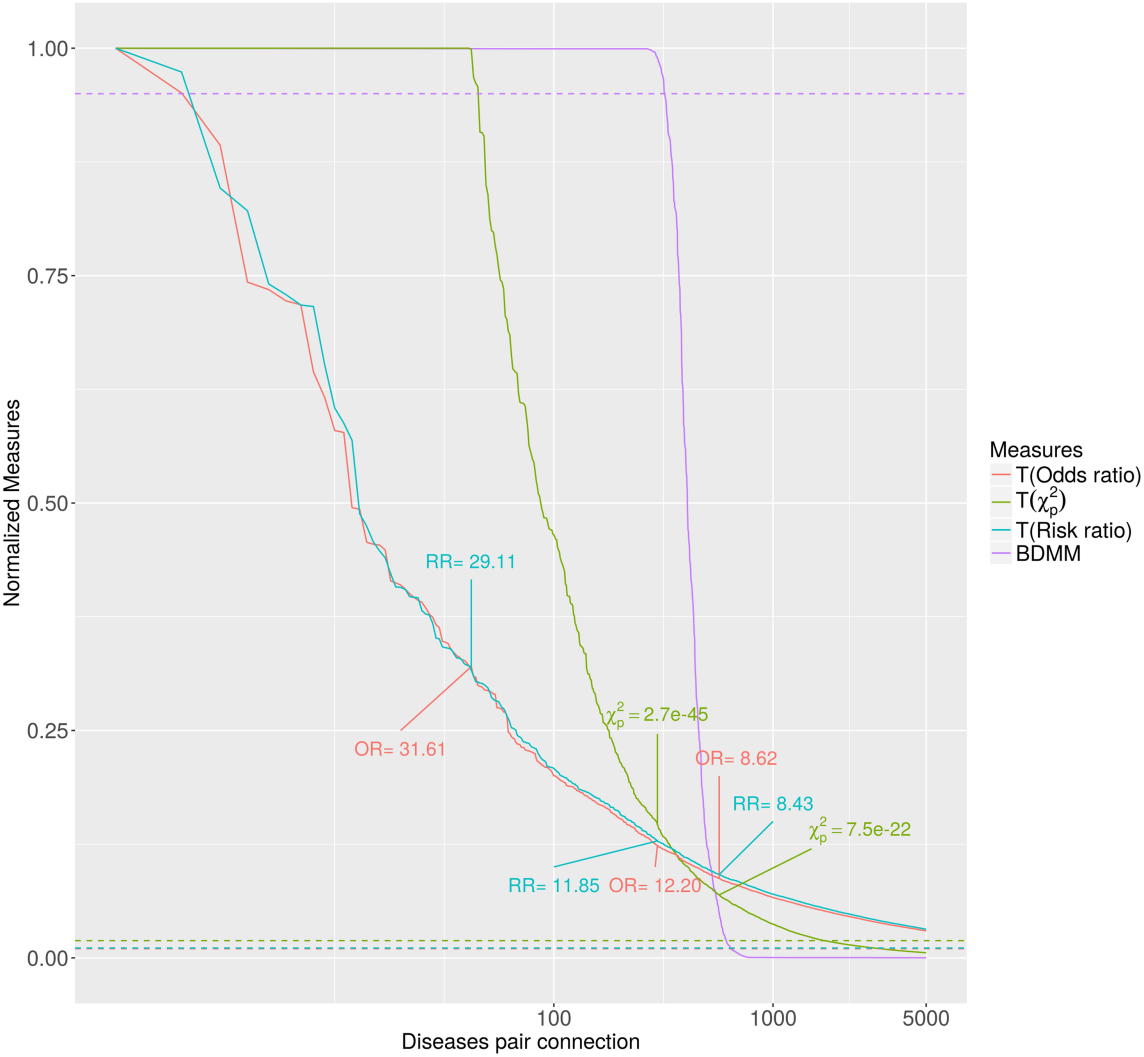
The top 5000 connections based on different measures. The BDMM edge posteriors (purple) together with the transformed connection values (odds ratios: red, risk ratios: blue and *χ*^2^ p-values: green). Dashed lines show the cut-off thresholds for the different measures. The given values show the original OR, RR and *χ*^2^ p-values.

We also investigated the sufficiency of the sample size on different approaches. Fig 4 shows characteristics of the most significant results in each approaches. We transformed the different scores to the [0, 1] interval to make them comparable (see Materials and methods). Descriptors and statistics are available on the web (Co=MorNet: bioinformatics.mit.bme.hu/UKBNetworks), also as an interactive tool to visualise networks of direct and mediated connections of selected diseases.

**Fig 5:**
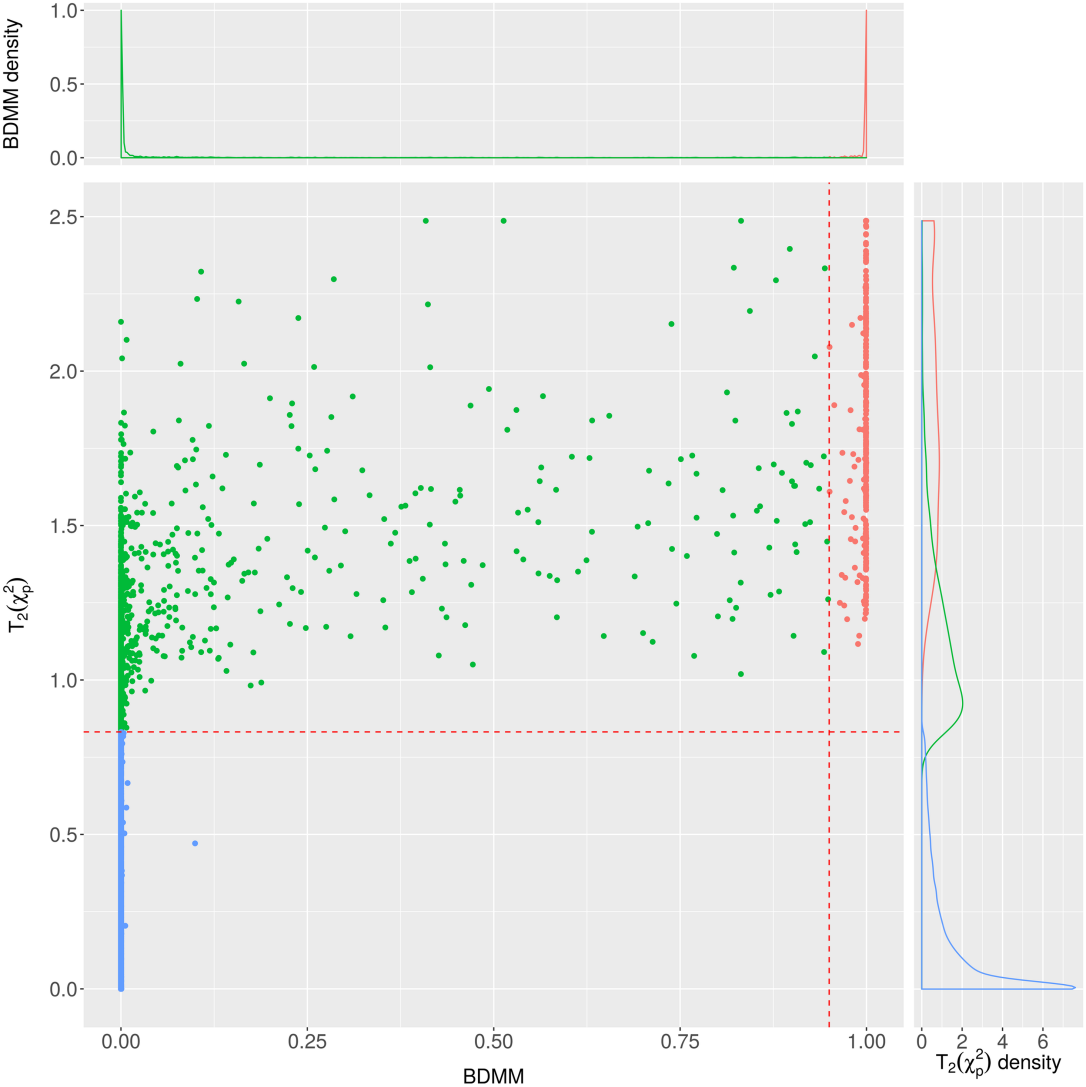
The scatterplot of BDMM posteriors together with transformed Bonferroni-corrected *χ*^2^ p-values. The different colors show the connections which are significant by both methods (red), significant only based on parametric association (green) and not significant (blue). For the BDMM we used a threshold of 0.95 whereas for the *χ*^2^ test a 0.05 threshold after Bonferroni-correction was applied. The density plots are scaled to 1 separately.

**Fig 6:**
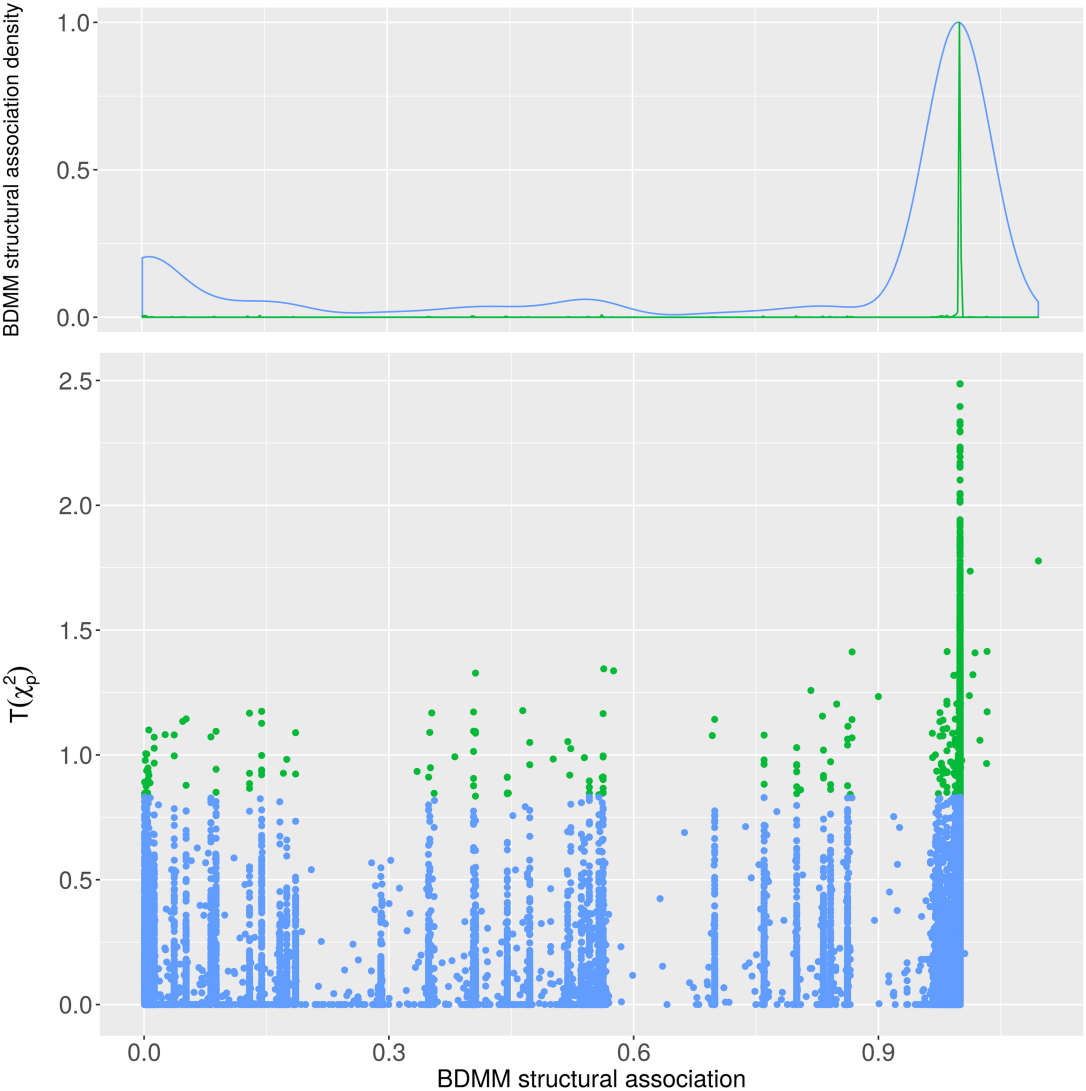
The BDMM structural association and the transformed *χ*^2^ p-values. Disease-disease connections shown with less than the 0.95 BDMM edge posterior. Green dots represents the connections significant by the *χ*^2^ independence test (p-value<0.05 after Bonferroni correction), whereas blue dots denotes the remaining connections.

The examination of the filtering capacity of the BDMM approach confirms that the BDMM edge posteriors strongly differentiate the direct connections from the mediated ones, e.g. there are only a few disease pairs which have a BDMM edge posterior between 0.05 and 0.95 (Fig 5). For the definition of BDMM edge and BDMM (structural) association relations see Table S1 in S1 Appendix. On Fig 5, we mark the direct comorbid connections with high BDMM edge posteriors (> 0.95) in red, showing that all of them have significant Bonferroni-corrected *χ*^2^ p-values. Additionally, focusing on a subset of relations with BDMM edge posterior less than 0.95, we examined the BDMM association posteriors versus the parametric association (see Fig 6). It confirms that almost all such strongly significant parametric association has high BDMM association posterior. Note, that relations with high BDMM edge posterior have high BDMM association posteriors as well. Furthermore, Fig 6 also illustrates that BDMM association posterior indicates many more structurally distant -presumably parametrically weaker- associations which cannot be inferred by parametric association tests such as Bonferroni-corrected *χ*^2^ p-values.

**Fig 7:**
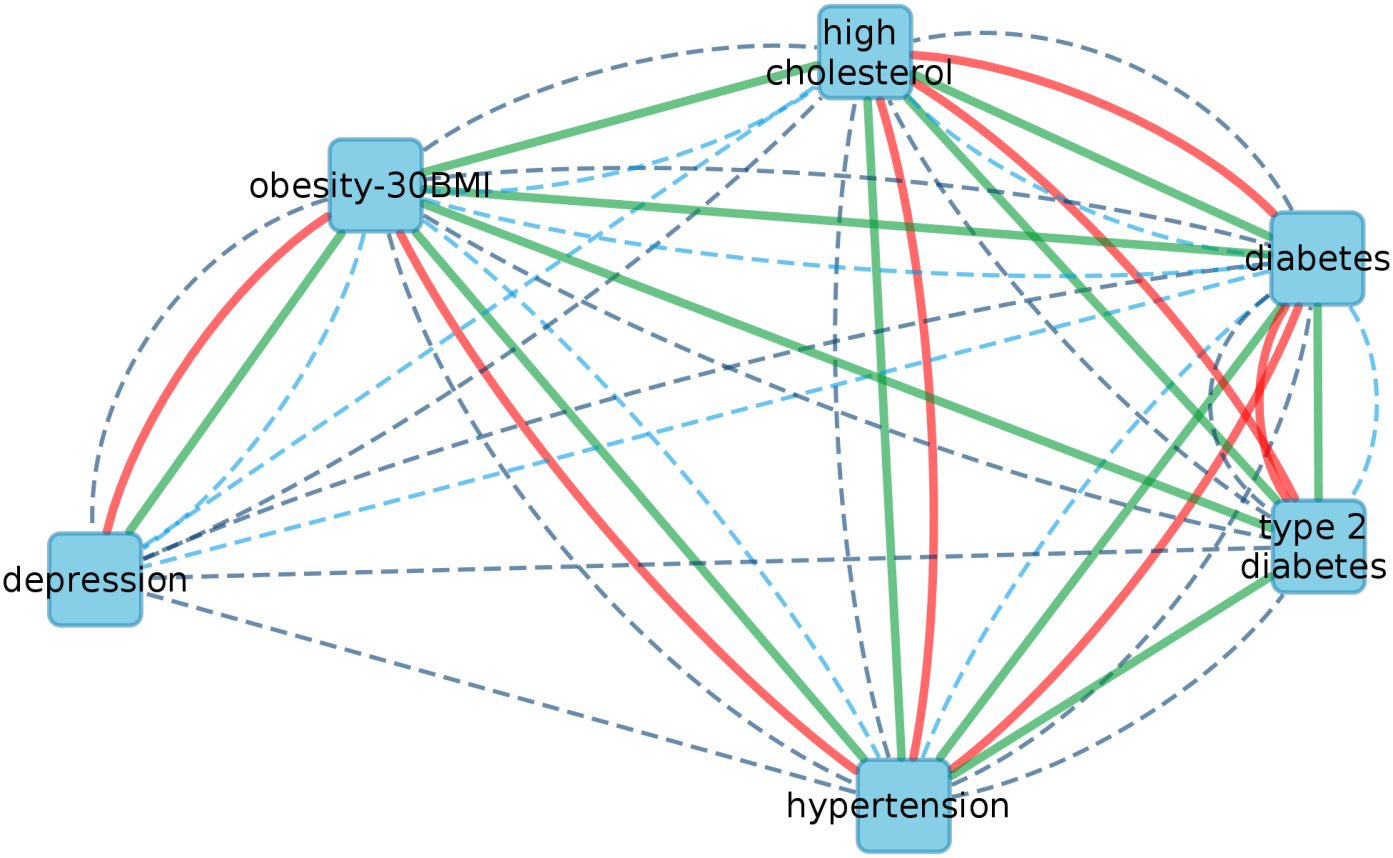
Subnetworks of pairwise and systems-based relations from both epidemiological and molecular levels. Solid lines show the systems-based relations: separation scores with negative values in red and BDMM Pr> 0.05 in green (for exact values see Table S3 in S1 Appendix. Note, that all of them except two relations have posteriors above 0.999). Dashed lines represent the pairwise associative metrics: relative risk with 95% confidence interval excluding 1 in dark blue (for details see S2 Dataset) and genetic overlap with hypergeometric distribution p-value below 0.05 in light blue.

To evaluate the genetic relevance of direct versus indirect comorbidity relations, we extended the epidemiological-level analysis with molecular-level approaches using a genetic overlap [3] and the interactome-based separation score [16]. This analysis included depression, metabolic syndromes and hypertension, see Fig 7, S2 Fig and Table S3 in S1 Appendix.

The genetic overlap measures for comorbidities were computed based on manually curated databases, using the DisGeNet [44] and NCBI PheGenI [45] (for details, see S1 Appendix, for a related earlier work, see [2]). The interactome-based connectivity/separation scores for comorbidities were computed by the supplied method and data from Menche et al. [16] (see S1 Appendix).

### Comorbidities and co=morbidities of depression

Beside of the investigation of direct and indirect status of comorbidities and the cross-comparison of pairwise and BDMM measures, we performed a more detailed medical evaluation of BDMMs on psychiatric and metabolic disorders, especially on depression and its comorbidities.

#### Comorbidities of depression based on the scientific literature

We explored the relations between the set of 426 potential comorbid disorders of depression using shallow text-mining methods with 2 different corpora: relevant PubMed abstracts (http://www.ncbi.nlm.nih.gov/pubmed) and PMC articles(http://www.ncbi.nlm.nih.gov/pmc/). The co-occurring disease pairs with depression, defined by MeSH terms for major depressive disorder (see Materials and methods), are listed in S1 Dataset. As expected, the well-known psychiatric comorbidities of depression, such as bipolar disorder, schizophrenia and anxiety disorders showed the highest rank in both corpora. Regarding metabolic disorders, diabetes mellitus ranked highest, followed by obesity. The top ranks of somatic comorbid disorders included neurodegenerative disorders, dementia, fibromyalgia, chronic fatigue, Parkinson’s disease, and migraine (for full list, see S1 Dataset). In addition, cerebral and cardiovascular disorders (e.g. heart disease, hypertension) ranked high in the list of disorders based on co-occurrence with depression.

#### Comorbidities of depression using non-systems based methods

Pairwise methods, identified anxiety and other psychiatric conditions as the strongest comorbidities in line with the literature (schizophrenia n = 94 and dementia n = 17 were too infrequent to be included in this analysis). These disorders were followed by fibromyalgia, and migraine similarly to the text-mining results. Cardiovascular (e.g. hypertension) and metabolic disorders (e.g. obesity, high cholesterol, and diabetes) were also identified as comorbid disorders with depression. To focus on the factors influencing our target disorder depression, we also applied logistic regression without interactions as a standard epidemiologic tool. S3 Dataset contains the coefficients and p-values of the significant disorders corresponding the UK Biobank dataset. There were many similarities with the pairwise *χ*^2^ method but whereas obesity and high cholesterol were associated to depression, diabetes or other cardiovascular disorders were not. This suggests that logistic regression may exclude mediated comorbidities (e.g. cardiovascular disorders may seem comorbid with depression because obesity and high cholesterol independently increase the risk of both depression and cardiovascular disorders). Painful disorders such as osteoarthritis, spondylitis, and back problem showed significant influences on depression in the logistic regression analysis, which are also frequently investigated conditions in association with depression [35, 36].

#### Co=morbidities of depression using systems-based multimorbidity maps

**Fig 8:**
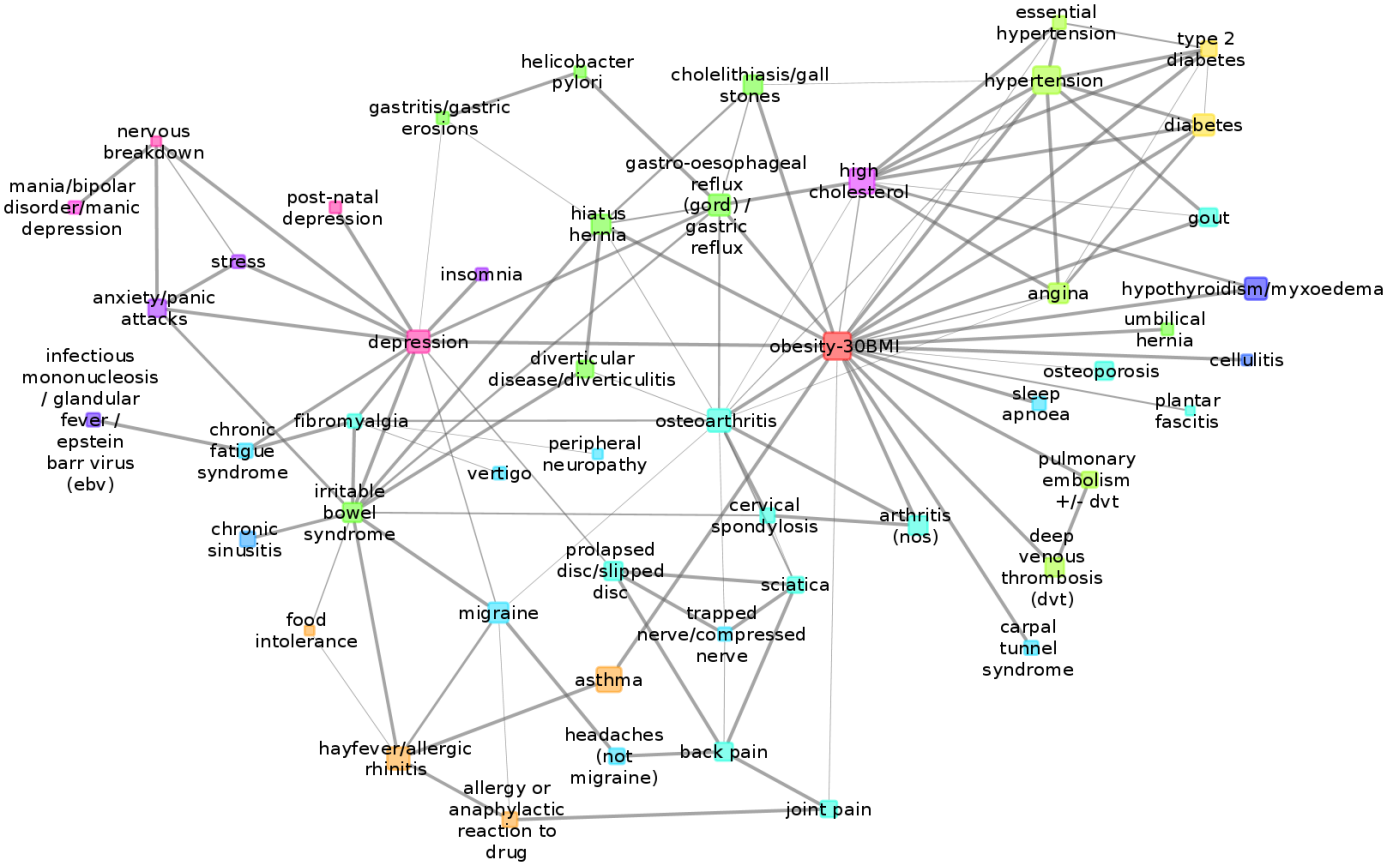
The Bayesian direct morbidity map around depression containing the neighbors of depression at maximum distance of two. The thickness of lines denote the strength of the link for being a member of the network above the cut-off threshold of posterior probability Pr = 0.05. Sex and age are not shown in the figure as these nodes would bring along many nodes which are related to depression only through these. Colors indicate higher level ICD-10 categories.

BDMM of depression can be seen in Fig 8, which shows the co=morbidities of depression and the mediated comorbidities, namely which disorders are not directly comorbid with depression but comorbid through another direct condition.

In line with the scientific literature and recent genetic studies, our results demonstrated that anxiety related disorders (anxiety, panic attack, stress, nervous breakdown), and postnatal depression are highly comorbid with depression (with posterior probability Pr = 0.99) suggesting common biological background. We noted that mania/bipolar disorder/manic-depression was not co=morbid with depression, suggesting distinct pathogenesis, but both were directly associated with nervous breakdown. Although nervous breakdown is not a psychiatric diagnosis, lay people often refer to it when in the face of stressful situation they could not function properly in everyday life due to excessive anxiety and depressive symptoms; it can be regarded as an indication of their severity. Similar stable co=morbidities emerged between psychiatric disorders when reported depression was replaced in the BDMM by a derived depression category based on the Mental Health Questionnaire data [46] (see S1 Appendix and S3 Fig). Taking into account the severity of depression provided further evidence that mania/bipolar disorder/manic-depression, nervous breakdown and fibromyalgia are co=morbidities with severe recurrent depression [46], while obesity was co=morbid with recurrent moderate depression. Note, that anxiety showed strong co=morbidity with all depression subcategories (see S1 Appendix and S4 Fig).

#### Effect of onset time on co=morbidities of depression

**Fig 9:**
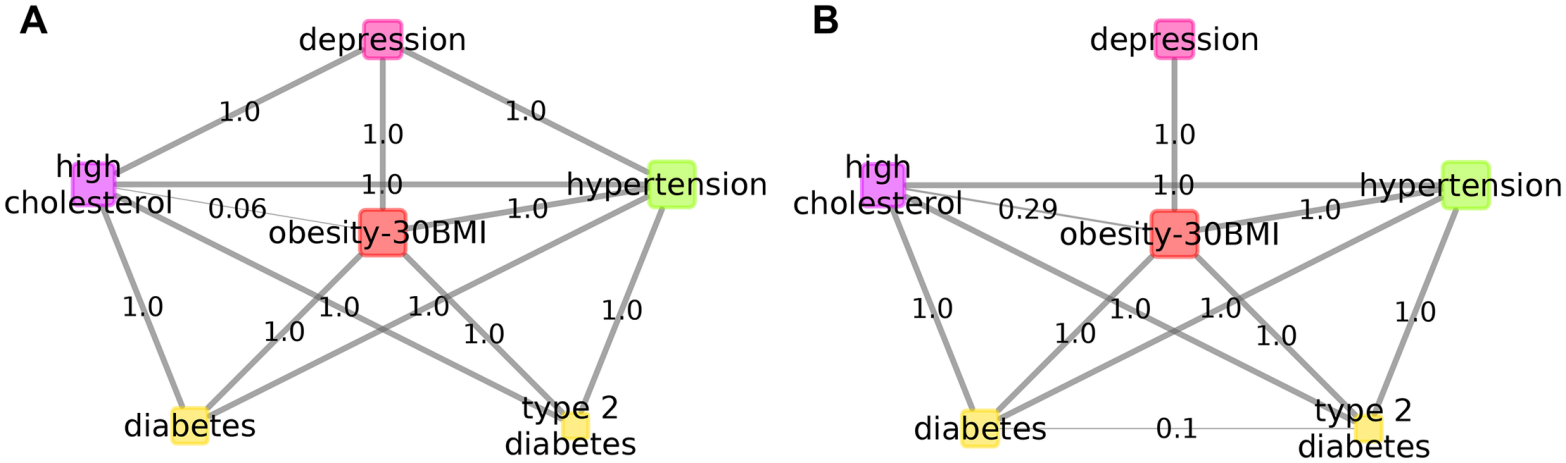
BDMM onset time dependence. BDMM of depression and metabolic disorders and hypertension. a: demonstrates co=morbidities with onset time prior to depression, while b. showing the BDMM computed on the full data regardless of onset time.

To explore the effect of onset time, we applied the same methodology on a filtered dataset, which excluded instances of diseases that occurred after the onset of depression. Anxiety related disorders and postnatal depression remained highly comorbid with depression (with posterior probability Pr = 0.99). Obesity in the full dataset showed a firm (Pr = 0.99) comorbidity with depression regardless of which depression definition was used (Fig 9 and S3 Fig). Although the time of onset of obesity is unknown, we retained it as a variable preceding depression, and found similar posteriors in both models suggesting its direct relevance for and possibly biological overlap with depression. Interestingly, both high cholesterol and hypertension were indirectly associated with depression in the full analysis, but showed strong direct comorbidity with depression when occurring before depression suggesting biological overlap in a subset of patients, although the effect of environmental factors, such as lifestyle, diet or medication may further increase the risk of depression in this subpopulation. Regarding diabetes and type 2 diabetes our results showed that these are not co=morbid disorders with depression but more likely obesity, high cholesterol, hypertension mediate their high co-occurrence with depression (Fig 9).

Another interesting cluster of comorbid disorders are irritable bowel syndrome (IBS), fibromyalgia (FM), chronic fatigue syndrome (CFS) and migraine; all of them showed strong link with depression based on the text-mining data (S1 Dataset). The BDMM showed that they are strongly co=morbid with depression in the full analysis but when the onset is before depression these strong relationships were absent. (S5 Fig). The high posteriors in the full analysis, and their sharp decrease in the restricted analysis may indicate that these disorders are heterogeneous themselves: in some subgroups of disorder the symptoms are part of the depression phenotype with high biological overlap but other subgroups maybe independent of depression or adversities that non-specifically predispose to depression. Similar patterns emerged for insomnia, gastro-oesophageal reflux (gord) / gastric reflux, prolapsed disc/slipped disc, and gastritis/gastric erosions suggesting that in some circumstances they are directly related to depression but in others they are independent of depression (see web tool, Co=MorNet:bioinformatics.mit.bme.hu/UKBNetworks). When we changed the depression definition to the one defined by low mood and anhedonia, ignoring somatic symptoms [46], only chronic fatigue and fibromyalgia showed co=morbidity with depression, especially with severe depression, suggesting that IBS, migraine, and other above mentioned somatic disorders may have specific relevance for depression dominated by somatic symptoms (for further detail see S3 and S4 Figs and S1 Appendix).

## Discussion

Large-scale cohort studies collecting life style, environmental, physiological, clinical and molecular level data, provide unprecedented opportunity for understanding health, pre-disease states, multimorbid conditions and progressions, especially to use epidemilogical level information to complement molecular level discoveries [2, 3, 8–13, 47]. However, the hypothesis-free, omic level use of comorbidites is hindered by multiple factors, such as by errors and biases in disease coding and collection of clinical information and by confounders like therapies, drug consumption or paradoxically the shared genetic factors themselves. A further imminent challenge is the presence of disease-mediated or disease-confounded comorbidity relations, i.e. indirect with respect to the selected diseases. In network science, algebraic solutions were proposed to attenuate indirect relations [19], but these solutions do not take into account the complex system of probabilistic dependencies between morbidities. We proposed to use probabilistic graphical models, specifically Bayesian networks to discriminate direct and indirect relations, because their semantics perfectly captures this aspect [43, 48, 49]. Indeed, the exact probabilistic treatment of a direct relation with respect to a given set of variables relies on the practical assumption of stability [48, 50], less demanding than assumptions for a causal interpretation [51].

Based on this probabilistic foundation of directness (“BDMM edge”), we constructed BDMMs using a subsample from the UK Biobank cohort to make visible the essential relations generating the multimorbidity patterns. We investigated BDMMs internally by comparing the BDMM edge posteriors and the structurally implied associative relations (the BDMM structural association posteriors), we cross-compared BDMMs with wide-range of pairwise comorbidity measures, we compared BDMMs against genetic overlap and interactome-based comorbidity scores. Additionally, we examined the comorbidity relations of psychiatric and metabolic disorders, specifically for depression in BDMMs.

### Co=morbidities versus structural and parametric associations

First of all, results indicate that the UK Biobank dataset is sufficiently large for the construction of BDMMs for this variable set, as the BDMM edge posteriors are peaked at 0 and 1 (see upper part in Fig 5), indeed, with the thresholds 0.05 and 0.95 we can efficiently separate the statistically significant comorbid relations to direct (co=morbid) and indirect relations. Notably, BDMMs eliminated more than 80% of comorbidity relations as indirect ones (320 direct connections from 1714 candidate relations). Interestingly, a recent work using a priori disease categories for restricting comorbidity relations, controlling for confounding with incidence characteristics and using two independent data sets similarly reported nearly 90% elimination ratio [12]. Note that all co=morbid connections were also confirmed by standard statistical methods (the right-lower quadrant is empty in Fig 5), which implies the technical condition that the distribution of the BDMM posteriors is stable [48–50]. In Fig 6 we further evaluated the connections with significant Bonferroni-corrected *χ*^2^ p-values but below the 0.95 edge posterior threshold. This shows that most of these connections have high BDMM structural association posterior, which suggest that BDMM indeed filtered mediated and confounded relations. There are 31 connections which have a significant pairwise association score but no structural association (BDDM structural association posterior < 0.1). These disorder pairs rarely occur together in patients (mean co-occurrence:8.45, with standard deviation: 4.9, and quantiles: 5, 7, 10.5 for the 25%, 50% and 75% respectively). In case of the *χ*^2^ test we applied Yates’ continuity correction but for such weak connections even with the large UK Biobank dataset the BDMM approach was not able to catch that weak dependency structure.

### Co=morbidities: Shared genetics and the molecular interactome

Our results demonstrated that the interactome-based score provided similar maps as BDMM co=morbid diseases, in sharp contrast to the associative genetic overlap scores which followed the pattern of the pairwise disease relative risk (see Fig 7, S2 Fig and Table S3 in S1 Appendix). Note that the interactome-based score and co=morbidity are analogous as both use a systems-based approach on different levels (molecular and epidemiologic level respectively). For detailed description of the molecular level methods and results see S1 Appendix and [2, 16].

### Psychiatric disorders as co=morbidities with depression

The high comorbidity between mood disorders and anxiety or stress related disorders is well known, and twin studies suggested that these comorbidities originated mainly from shared genetic risk factors [52, 53]. Our results showed another expected aspect, namely that this relationship is independent of the order of the onset of these disorders. This observation is in line with a longitudinal study which showed that generalised anxiety disorder (GAD) and major depressive disorder (MDD) are strongly comorbid with an equal probability of GAD or MDD occurring first or simultaneously suggesting they might not be distinct disorders [54]. Although overlapping genetic risk factors for anxiety and depression have not yet been identified, common genetic vulnerability has been found for other comorbid psychiatric disorders [32, 33, 55]. Our BDMM further indicate strong and stable co=morbidity between depression, anxiety, stress, postnatal depression and nervous breakdown, pointing toward interactome-level overlaps; this reinforces the need to find potential common biological mechanisms [56].

### Multimorbidity pattern of metabolic disorders and hypertension with depression

Epidemiologic studies repeatedly report high comorbidity between depression and metabolic disorders [57], depression and diabetes [58], depression and cardiovascular disorders [37], depression and hypertension [59, 60], and depression and obesity [61, 62]. However, there have been several contradictory results, and this suggests a more complex relationship. Indeed, recent GWAS results found no shared genetic risk between these disorders and depression [55]. Nevertheless, based on the UK Biobank cohort data, obesity was co=morbid with depression while cardiovascular disorders, hypertension, high cholesterol and diabetes, including type 2 diabetes were only indirectly related to depression. When we excluded occurrences after the onset of depression the direct relationship between obesity and depression remained as expected but entirely new links with high posterior probability emerged suggesting a strong relationship between the consequences of metabolic syndromes and depression. Studies of the genetic relationship between obesity and depression suggest that atypical depression, characterised by increase in appetite and weight, is associated with genetic risk factors and polygenic risk scores of increased BMI and triglycerides, while typical depression, with decreased appetite and weight, show more similarities with other psychiatric disorders [63, 64]. Thus, in line with our results comorbid obesity and metabolic disorders may identify a specific subtype of depression with a distinct biological background. Caution is required in inferring shared biology for co=morbid disorders given our current lack of knowledge about relevant GxE interactions. Although metabolic disorders are only co=morbid with depression when they precede it, a variety of non-biological associations of mediators may be at play. We cannot currently exclude the possibility that lifestyle factors, such as diet, physical activity and stress, or medication used to treat hypertension, hypercholesterolaemia and obesity may contribute to the later development of depression [65]. As a specific example, a previous study demonstrated that current psychological distress amplified the effect of genetic risk of high BMI [66]. Patients with increased genetic risk to become overweight showed worse physical outcome (higher BMI), and quite probably more comorbid psychological symptoms, when life stress was present. Furthermore, it has been reported that statins, drugs with cholesterol-lowering effect, have antidepressant effect in patients with comorbid depression and coronary artery disease while the same drugs can have pro-depressive effect or no effect on depression when comorbidities and depression subtypes were not taken into account [67].

### Multimorbidity pattern of IBS, FM, CFS and migraine with depression

Migraine [34], IBS [68], FM [69], and CFS [70] are highly comorbid with depression based on epidemiologic studies. It is therefore puzzling that they involve different etiological mechanisms. In addition, their symptoms often overlap making it difficult to apply diagnostic categories. We found that these disorders were not relevant when they occurred before depression but were highly co=morbid in the full analysis. The probable explanation is that in general, these disorders are related to consequences of depression and only specific subtypes of these disorders can be expected to have causal relations, e.g. shared biological background with depression. For example, a genetic risk score analysis demonstrated that migraine with comorbid depression was more genetically related to depression than to pure migraine, which suggests that migraine might develop as a consequence of different polygenic backgrounds [71]. Similarly, a large general population cohort study confirmed that FM, CFS and IBS increase the odds of depression and anxiety but that most patients who suffer from FM, CFS and IBS have no mood or anxiety disorder [72].

### Limitations

One of the main limitations is that all disorders were self-reported, although trained nurses evaluated and corrected all entries during face-to-face interviews. The second one is that the applied treatments or medications were not included in the analysis which could highlight comorbidities due to the side effect of treatments. We will address this problem in follow-up studies. Note that we only used a subset of the UK Biobank dataset selecting those participants who filled out the Mental Health Questionnaire and provided online dietary information, which may introduce confounding through selection bias. However, limiting our study to this subpopulation enabled us to test different definitions of depression and will allow us to connect this comorbidity network to relevant environmental risk factors.

### Conclusion

The use of large-scale health data sets, such as the UK Biobank dataset hold the promise of complementing and guiding the molecular level research of complex diseases. Adopting an intermediary approach between statistical association analysis and causal discovery we investigated the use of Bayesian networks in the Bayesian model averaging framework to explore direct probabilistic relations with respect to a given set of variables, i.e. to eliminate confounders and mediatory effects by a systems-based approach.

We demonstrated the applicability of BDMMs, especially their principled capability of discriminating direct and indirect comorbidities. In summary, PGMs offer maximally sparse dependency models and utilize the omic nature of the epidemiologic data jointly modelling all the morbidities; while the Bayesian approach through posteriors provides an explicit representation for the uncertainties in a dataset. Thus the Bayesian direct morbidity maps provide sparse, systems-based, omic-wide perspectives.

From a clinical perspective, our results also highlight that the direct and indirect subtypes of comorbidities support a finer biological interpretation, namely an interactome-based detailed interpretation using molecular mechanisms corresponding to direct relations, whereas genetic overlap using associative gene sets may only reflect indirect comorbidities. In addition, re-running the analysis by including only instances of disorders which preceded depression, we delineated comorbid disorders of depression with more refined causal roles that could specify subgroups of depressed patients with more homogenous background.

The investigation of Bayesian direct morbidity maps also demonstrated, that even large-scale datasets such as UK Biobank, are still limited for non-ambiguous identification of complex dependency patterns such as multimorbidities [73]. However, the applied Bayesian statistical framework offers an automated, normative solution for the multiple hypothesis testing problem and the application of probabilistic graphical models in the Bayesian framework supports the versatile post-processing of the results and their efficient communication and sharing. The results of our research highlight the advantages of Bayesian systems-based modelling, which could be vital to cope with the growing heterogeneity of new health data sets containing full personal genetic information with high dimensional data about lifestyle, environmental factors and sequential decisions on drug therapies [8, 74].

## Materials and methods

### Databases

In the present study we used the UK Biobank (http://www.ukbiobank.ac.uk/ and [75] cohort where subjects’ chronic illness history together with onset age were ascertained by trained nurses during face-to-face interviews and were processed by experts resulting in 525 different disease categories. The investigated subset in this study consisted of 117,392 participants (female: 64,320; male: 53,072) who provided the extended Mental Health Questionnaire (http://biobank.ctsu.ox.ac.uk/crystal/label.cgi?id=100060) and the online diet questionnaire data together with the extensive baseline dataset. We used the UK Biobank original disease categories with at least 1‰ prevalence in the selected subset, which resulted in n = 247 diseases including depression (n = 6040). In addition, we coded obesity in cases where BMI were equal or greater than 30*kg*/*m*^2^, for further analyses. For statistical analysis, sex was included into the data set, and age was binned into 3 equal frequency categories with thresholds 60 and 68 years. Then we applied the different pairwise measures and logistic regression together with Bayesian systems-based modelling to compare the models computed on these datasets (see below and in S1 Appendix). To investigate the effect of disease onset, self-reported disease onset data was used to filter the dataset. 6,040 patients affected by depression, provided onset data whose comorbid illnesses were eliminated if they occurred after the onset of depression. After removal of diseases with prevalence less than 1‰ the dataset contained 241 diseases. We extended the dataset with sex, age and BMI-based obesity. The data were analysed using same statistical methods as with the non-filtered dataset. To test the stability of comorbid relationships with depression we also used an alternative depression definition instead of self-reported depressive disorder. Depression and its severity was defined by the Mental Health Questionnaire data [46], for definition see S1 Appendix. These alternative depression categories were analysed with Bayesian systems-based modelling.

### Statistical methods

We applied text-mining and conventional statistical methods to explore comorbid relations, see S1 Appendix. For these computations we used in-house written R scripts together with the statistical programs included in the stats package of R [76]. To overcome the limitations of these conventional methods, we applied a Bayesian network Markov Chain Monte Carlo (BN-MCMC) method to explore the overall system of dependencies-independencies, visualized as an undirected graph with weighted edges [20, 21, 23–25, 42]. The weighted edges correspond the a posteriori probabilities (Pr) of direct, nonmediated “co=morbidity” relations, the weights are in the [0, 1] interval and the higher values show stronger relationship. The systems-based approach using Bayesian networks prunes the indirect, mediated connections between morbidities, thus resulting in a sparse co=morbidity map compared to pairwise association networks (for detailed description of the method and for further types of dependency relations, see S1 Appendix and Table S1 in S1 Appendix).

#### Transformations

We transformed odds ratios, risk ratios and *χ*^2^ p-values to the [0, 1] interval and we inverted the scale of *χ*^2^ p-values as follows.

$$\begin{array}{c} T\left(\chi_{p}^{2}\right)=\{\begin{array}{cc} -\frac{log_{10}\left(\chi_{p}^{2}\right)}{ld{\left(p\right)}^{*}+1} & \text{if}\;\chi_{p}^{2}\neq 0\\ 1 & \text{if}\;\chi_{p}^{2}=0, \end{array} \end{array}$$ (1)

where $\chi_{p}^{2}$ is the p-value of the *χ*^2^ test and $ld{\left(p\right)}^{*}=\max_{\chi_{pi}^{2}>0}\left(-log_{10}\left(\chi_{pi}^{2}\right)\right)$. In this paper, we refer to $T(\chi_{p}^{2})$ as *parametric association*.

$$\begin{array}{c} T\left(\mathrm{OR}\right)=\{\begin{array}{cc} \frac{\mathrm{OR}}{{\mathrm{OR}}^{*}} & \text{if}\;1\leq \mathrm{OR}<100\\ \frac{1}{\mathrm{OR}\cdot {\mathrm{OR}}^{*}} & \text{if}\;0.01<\mathrm{OR}<1\\ \text{ignored} & \text{otherwise}, \end{array} \end{array}$$ (2)

where ${\mathrm{OR}}^{*}=\max_{0.01<\mathrm{OR}<100}\left(\mathrm{OR},\frac{1}{\mathrm{OR}}\right)$.

$$\begin{array}{c} T\left(\mathrm{RR}\right)=\{\begin{array}{cc} \frac{\mathrm{RR}}{{\mathrm{RR}}^{*}} & \text{if}\;1\leq \mathrm{RR}<100\\ \frac{1}{\mathrm{RR}\cdot {\mathrm{RR}}^{*}} & \text{if}\;0.01<\mathrm{RR}<1\\ \text{ignored} & \text{otherwise}, \end{array} \end{array}$$ (3)

where ${\mathrm{RR}}^{*}=\max_{0.01<\mathrm{RR}<100}\left(\mathrm{RR},\frac{1}{\mathrm{RR}}\right)$.

## Supporting information

S1 Appendix Supplementary material. Detailed description of methods.
(PDF)

S1 Fig Comparison of different comorbid network approaches. (TIF)

S2 Fig Comparison of different molecular- and epidemilogic level statistics. (TIF)

S3 Fig Bayesian direct multimorbidity map (BDMM) with the alternative single binary depression indicator. (TIF)

S4 Fig Bayesian direct multimorbidity map (BDMM) using multivariate depression analysis. (TIF)

S5 Fig Bayesian direct multimorbidity map (BDMM) for depression, irritable bowel syndrome, chronic fatigue, fibromyalgia and migraine. (TIF)

S1 Dataset The results of text-mining for both corpuses, PMC and PubMed. (XLSX)

S2 Dataset The results of the classical statistical measures for all pairs of factors including sex and age. (XLSX)

S3 Dataset Results of logistic regression. (XLSX)

S4 Dataset The Bayesian direct multimorbidity map (BDMM) results. (XLSX)
