# Supplementary material for: Comorbidities in the diseasome are more apparent than real: What Bayesian filtering reveals about the comorbidities of depression
Source: PLoS Comput Biol. 2017 Jun 23;13(6):e1005487. doi: 10.1371/journal.pcbi.1005487 (PMC5507322; doi:10.1371/journal.pcbi.1005487)
Supplement: S1 Appendix — Detailed description of methods. (PDF) [file pcbi.1005487.s001.pdf]

Supporting Information

Comorbidities in the Diseasome Are More Apparent Than Real: What Bayesian Filtering Reveals about the Comorbidities of Depression

Peter Marx et al.

Text-mining

We collected PubMed abstracts annotated with MeSH terms for major depressive disorder and full PMC articles to construct two different corpora. The PubMed search resulted in a corpus of 96,968 entries 8,902 containing the 'major depressive disorder' expression. The PMC articles were segmented into sentences to build the second corpus. 'Major depressive disorder' occurred 1,482 times in the 20,865,176 sentences. Each diseases with MeSH annotation was counted in both corpora as well as co-occurrence of disease pairs. The following disorders in the corpora were accounted as depression: atypical depressive disorder, major depressive disorder, moderate recurrent major depression, endogenous depression, mental depression, psychogenic depressive psychosis, chronic depressive disorder, postpartum depression. These depressive disorders were found in both corpora except moderate recurrent major depression which appeared only in the PMC corpus.
The results of text-mining can be found in Supplementary Dataset S1.

Additional information to the applied methodology for network computation

Earlier works often focused on bivariate comorbid models and used a latent factor approach to describe the connection between two diseases [1, 2]. These bivariate models were extended to examine the connections between multiple disorders. A detailed introduction to bivariate and multivariate psychological comorbid models is provided by Krueger et al. and Middeldorp et al. [1, 2]. Multivariate models have typically used pairwise measures or structural equation modelling (SEM) to model comorbidity. Pairwise measures are easy to implement but cannot take into account confounding factors and often overestimate specific relations: for example relative risk is known to overestimate comorbidity of rare diseases. Structural equation modelling overcomes this problem but uses models defined by the user. Logistic regression (LR) is another commonly used tool to examine the relationships between a target disease and multiple other disorders. LR has the advantage of testing for interactions between covariate disorders on the target disease. Conversely, LR defines the interactions only between the target disorder and the others and cannot define causality. In "reality", comorbid disorders work as a dynamic, feed-back multi-morbid network (Fig. S1 A) which can be assessed by modelling complex medical datasets. However, pairwise measures (Fig. S1 B) introduce many unreal connections, or 'edges in the language of graph network theory, between indirectly connected disorders ('nodes') and cannot define causality between the diseases. In models based on logistic regression, only the directly connected subset of the nodes can be identified (Fig. S1 C). The best approximation of the real network structure can be achieved by applying probabilistic graphical modelling (PGM) approach (Fig. S1 D) which could potentially define

the dependencies between the disorders, although it fails to find feed-back loops. The applied Bayesian systems-based methodology is a specific subtype of PGMs. To describe the investigated disorders together with sex and age we use the expressions 'variables' or 'variable set' in this study.

Figure S1 Comparison of different comorbid network approaches. Circles denote diseases, and the red circle with T used as the target disease. **A.** The real, dynamic network containing feed-back loops **B.** Comorbid network computed by pairwise statistical measures. The darker lines are the real direct connections, which secondarily generate many unreal edges (lighter lines). **C.** Multivariate model, e.g. logistic regression. Only the neighbors of the target node are identified. **D.** Bayesian systems-based comorbid network, which is able to detect direct connections between diseases.

Classical statistics

We computed odds and relative risk with 95% confidence intervals, the mostly applied epidemiologic tools which makes our results comparable to others findings. Furthermore we used mutual information, Pearson's correlation coefficient and Fisher's exact test to examine disease relationships. As it is not straightforward to interpret a comparison between probabilities and odds we used Chi-squared statistics with the Yates' continuity correction to enumerate the strength of a connection in the co-morbid network. Logistic regression served as a tool for searching factors in depression. For these computations we used in-house written R scripts together with the statistical programs included in the stats package of R [3].

The results of these methods can be found in Supplementary Dataset S2 and S3.

Construction of Bayesian direct multimorbidity map (BDMM)

Probabilistic graphical models (PGMs) provides a formal, structural representation for the set of multivariate dependencies and independencies over morbidities, where each morbidity (disease) is represented by a graph node [4, 5]. A popular class of PGMs, Bayesian networks, uses directed acyclic graphs to represent multivariate dependencies and conditional independencies [6]. Within a general Bayesian framework for the quantitative dependencies, this representation is sound and complete with respect to its probabilistic interpretation, meaning that the represented multivariate dependencies and independencies are exactly the dependencies and independencies present in the distributions compatible with the graphical representations (for mathematical details, see e.g. [7]). Bayesian networks also offer a rich language for representing causal relations, see e.g. Table S1, but we use only the probabilistic interpretation and corresponding relations, such as direct dependence represented as an edge (link), to avoid further assumptions about the data and the domain [6, 8].

The Bayesian inference over structural properties of Bayesian networks was put forward decades ago [9], together with the first MCMC scheme [10]. This approach was successfully applied to characterize a complete domain using a posteriori probabilities of pairwise relations (see e.g [11-14]), and it was also useful to characterize the relevance of explanatory variables for a given target [11, 15-18]. Assuming complete data, Cooper-Herskovits parameter priors with virtual sample size 1 and uniform prior over structures with maximal 6 parental set size, the posterior of the structure G $P(G|D)$ can be computed efficiently [9]. The a posteriori probability of direct dependency between variables X and Y is defined as a marginal in Bayesian model averaging by the following sum

$$P(\text{Co} = \text{morbid(X, Y)} \mid D) = \sum_{G} P(G \mid D)(1(Edge(X, Y, G)) + 1(Edge(Y, X, G)))$$

where 1(.) denotes the indicator function, which gives 1 if the property holds in G and 0 otherwise. The direct dependency posteriors, i.e. edge posteriors, are estimated using a DAG-based Markov Chain Monte Carlo simulation [19]. The probability to apply different DAG operators in the proposal distribution was uniform, the length of the burn-in was 5×10^5 and the length of the sample collection is 1.5×10^6. To check the convergence of the MCMC simulation for the estimated posteriors, we calculated the Geweke Z-score and the Gelman-Rubin R-score (less than 0.1 and 1.1, respectively) and confidence intervals (less than 0.1) [20].

Supplementary Table S1 Different types of structural dependencies between morbidities using Bayesian network semantics. The co=morbidity relation is visualized in Bayesian direct multimorbidity maps (BDMM).

Connection type	Definition
**Direct comorbidity: co=morbidity**	
D1 parent of D2	There is a directed edge from D1 to D2
D2 parent of D1	There is a directed edge from D2 to D1
**Indirect or mediated comorbidity**	
D1 descendant of D2	There is a directed path from D2 to D1
D1 ancestor of D2	There is a directed path from D1 to D2
D1 confounded with D2	D1 and D2 have a common ancestor
**Pairwise association**	
D1 associated with D2 (pairwise association)	If there is an edge or directed path between D1 and D2 or D1 and D2 have a common ancestor
**No relationship**	
D1 independent of D2	There is neither direct nor mediated connection between D1 and D2

# Correspondance between BDMM and molecular interactome-based mapping

We used PheGenI [21] together with the curated DisGenet database [22] to find gene-disease associations. We extended this dataset with the data provided by Menche et al. [23] because a molecular level interactome-based map would allow a detailed investigation of the epidemiological relations [23]. Unfortunately, the UK Biobank uses in-house developed disease coding which is not linked to general taxonomies as Unified Medical Language System (UMLS) or to Medical Subject Headings (MeSH). Thus to provide interpretation of our multimorbid map a subset of the UK Biobank disease categories, containing depression, metabolic syndromes and hypertension, were translated into UMLS terms and the PheGenI search terms which were then used to define the disease-gene and disease-interactome associations (Supplementary Table S2).

Supplementary Table S2. The PheGenI search terms and UMLS identifiers used to define disease-gene associations within the PheGenI and the DisGeNet curated databases respectively.

UK Biobank disease	Corresponding UMLS terms	PheGenI search terms
depression	C1269683;C1837929;C1837529;C0011581	Depression; Depressive Disorder, Major
diabetes	C0011849;C0011854;C0011860;C0342277;C1832392; C1832474;C1832605;C1833218;C1838259;C1838260; C1838261;C1838262;C1852092;C1854125;C1857808; C1864068;C1866040;C1866041;C1866519;C2675472; C2675864;C2675865;C2675866;C2751697;C1832387; C1832544;C1842642;C1863594	diabetes mellitus; diabetes mellitus, type 1
high cholesterol	C0020443;C1839021;C1863512;C1863551;C1858233	cholesterol
hypertension	C0020538;C0085580;C1839021	hypertension
obesity	C0028754;C0028756;C0311277;C2675358;C2675659	obesity
type 2 diabetes	C0011860;C1832387;C1832544;C1842642;C1863594	diabetes mellitus, type 2

The software and the interactome network by Menche et al. [23] were used to compute the separation scores. Separation score describes the relationship of two diseases on the molecular level by utilizing the network of connected genes. The separation score characterizes the distance of gene sets associated with each of the two diseases. Larger, positive values represents distinct gene sets over the interactome whereas smaller, negative values represents more connected (closer) gene sets. In considering our results We used the threshold of 0 as in Menche et al. [23] for disease pair association. Next we compared the separation scores to relative risks and the BDMM posteriors. We present the results of this analysis in Supplementary Table S3.

Supplementary Table S3. The interactome-based separation score compared to the relative risk and the BDMM posterior probability.

Disease1	Disease2	Relative Risk	BDMM posterior	Separation score	Genetic overlap	Short name
obesity-30BMI	hypertension	2.051	0.999	-0.023	2.1E-8	Obes-Hypert
obesity-30BMI	diabetes	3.525	0.999	0.049	2.5E-5	Obes-Diab
obesity-30BMI	type 2 diabetes	4.331	0.999	0.117	0.242	Obes-T2D
obesity-30BMI	depression	1.557	0.999	-0.070	0.003	Obes-Depr
obesity-30BMI	high cholesterol	1.651	0.286	0.144	0.031	Obes-HighChol
hypertension	diabetes	5.196	0.999	-0.076	3.2E-8	Hypert-Diab
hypertension	type 2 diabetes	4.651	0.999	0.064	0.397	Hypert-T2D
hypertension	depression	1.190	0.025	0.043	0.234	Hypert-Depr
hypertension	high cholesterol	3.872	1.000	-0.027	0.154	Hypert-HighChol
diabetes	type 2 diabetes	0.510	0.099	-0.398	2.5E-62	Diab-T2D
diabetes	depression	1.149	0.000	0.049	2E-4	Diab-Depr
diabetes	high cholesterol	3.435	0.999	-0.121	2E-4	Diab-HighChol
type 2 diabetes	depression	1.702	0.000	0.053	0.382	T2D-Depr
type 2 diabetes	high cholesterol	3.389	1.000	-0.009	0.427	T2D-HighChol
depression	high cholesterol	1.243	0.000	0.062	0.04	Depr-HighChol

Moreover, we applied a common tool, hypergeometric distribution p-value to evaluate gene set overlapping [24]. This score describes that the size of the overlap between gene sets is how likely by chance (see Supplementary Table S3).

The evaluation of genetic overlap, interactome-based separation scores, the pairwise measure based relative risk and the BDMM posterior probability results can be seen in Figure S2.

Figure S2. Comparison of genetic overlap, interactome-based separation scores, pairwise measure (relative risk), and BDMM posterior probabilities in a subset of the UK Biobank disease categories.

Figure S2 shows the cross comparison of direct and associative metrics from epidemiological and molecular levels evaluating the hypothesis that the type of the metric (direct versus associative) is more influential than the level (epidemiological versus molecular) on their similarities. Specifically, the direct relation-based methods are consistent and sparse through the molecular (interactome-based separation score) and epidemiologic (BDMM based posterior probabilities)-levels.

First, we examined the comorbid disorders with high posterior and relative risk compared to the separation score (see Fig. S2 I-II). The separation score was positive in three cases (obesity-diabetes ‘B’, obesity-type 2 diabetes ‘C’, and hypertension-type 2 diabetes ‘G’). This probably reflects the missing interactions in the interactome as the epidemiological relations are well described in the literature. We can distinguish three different scenarios for the comorbid connections with moderate relative risk: (1) links with negative separation scores have high

BDMM posterior probabilities ('A' and 'D'); (2) while those with positive separation scores have almost 0 posteriors ('O', 'M', 'K', 'H'); (3) finally obesity and high cholesterol (link 'E') is the only case where the BDMM approach did not purify the connection but results in a moderate posterior of (Pr=0.29) and positive separation score. Figure S2 III-IV show the differences of relative risk and BDMM compared to the hypergeometric distribution p-values computed for genetic overlap. A marked difference can be observed for measures between type-2 diabetes and depression (‘M’), namely as the associative metrics (relative risk and genetic overlap) show strong connection on the contrary direct relation-based method, BDMM filters this link.

In summary, the compatibility of direct relation-based approaches suggest that these different level of information sources can be combined to reconstruct the complete multimorbidity map.

Results using the alternative depression definition

Smith et al. [25] computed alternative diagnoses of probable depression based on the Mental Health Questionnaire (http://biobank.ctsu.ox.ac.uk/crystal/label.cgi?id=100060) data filled out by the participants. Depression was coded positive if the patients selected „Ever felt depressed for a whole week” or „Ever disinterested or unenthusiastic for a whole week” and the „Episode lasted more than 2 weeks” and they have seen GP or psychiatrist for „nerves, anxiety, tension or depression”. Severity was determined as follows: Single Probable MD Episode if only 1 episode happened; Probable Recurrent MD (moderate) if more episodes reported but only GP was seen; Probable Recurrent MD (severe) if more episodes reported and psychiatrist was seen.

Using these alternative diagnostic categories we reanalyzed the data and compared to the multimorbidity map obtained by using the depression diagnosis ascertained during the face-to-face interviews. First we created a merged depression variable resulting in 21367 depressed participants (satisfied the definition above regardless of severity). In the second case we performed a multivariate analysis using the above depression severity categories with the following counts: single episode of depression: n=5421; recurrent moderate depression: n=10094; recurrent severe depression: n=5852. As many participants who reported depression during the face-to-face interview sessions were not categorized as depressed by the above questionnaire based methodology we added a fourth depression indicator for these patients (interview depression: n=2588). For this analysis we did not use the original interview based depression variable.

We used the BDMM method to compute the multimorbidity networks. In case of the merged depression category the results were similar to the original depression networks (see Fig S4). Depression has stable connections to other psychiatric disorders. The only difference is that bipolar depression has a direct relationship to depression using the merged variable (note that the alternative depression definition does not exclude subjects with bipolar features). The difference is bigger in case of somatic disorders. The merged variable has direct edges (co=morbidity) to obesity, fibromyalgia (FM), chronic fatigue syndrome (CFS) but the edges leading to irritable bowel syndrome (IBS) and gastro-oesophageal reflux (GORD) are missing. These are connected to depression indirectly through FM or obesity. Additionally in this analysis asthma is co=morbid with depression. Besides, migraine has an indirect relationship to depression through three paths namely: asthma--rhinitis--migraine, back pain—headaches (not migraine)--migraine, FM—IBS—migraine (see webtool, Co=MorNet: bioinformatics.mit.bme.hu/UKBNetworks).

Figure S3 Bayesian direct multimorbidity map (BDMM) with the alternative single binary depression indicator. Depression is the union of the different depressive disorders based on Mental Health Questionnaire data and defined by Smith et al [25].

In case of the multivariate depression analysis (see Fig S5) we examined the direct co=morbidities of the depressive disorders with different degree of severity. As expected the different types of depression form a complete sub-graph, because we derived them as mutually exclusive disease categories, which introduces technical connections between them (if one type of depression present in a patient another could not be). All psychiatric disorders have direct connections to one or more type of depression. The most remarkable observations are:

- Anxiety connects to all types of depression.
- Bipolar disorder binds only to recurrent severe depression.
- Stress is connected to recurrent depressions.

Four somatic disorders are co=morbid with at least one type of depression. Obesity has direct edges to recurrent moderate depression and interview depression. IBS connected only to the interview depression variable. FM has an edge to recurrent severe depression while CFS has edges to recurrent severe depression and interview depression.

Figure S4 Bayesian direct multimorbidity map (BDMM) using multivariate depression analysis. Different severity of depressive disorders were defined in [25].

Additional results for the onset time restricted analysis

Figure S5. Bayesian direct multimorbidity map (BDMM) for depression, irritable bowel syndrome, chronic fatigue, fibromyalgia and migraine in the full (A) and in the restricted (only disorders which onset was before depression, B) analysis.

List of Supplementary datasets

Supplementary Dataset S1 The results of text mining for both corpuses, PMC and PubMed. The table contains the occurrence of diseases together with co-occurrence with depression. Depression is defined by the union of different subtypes of depression, namely: atypical depressive disorder, major depressive disorder, moderate recurrent major depression, endogenous depression, mental depression, psychogenic depressive psychosis, chronic depressive disorder, postpartum depression.

Supplementary Dataset S2 The results of the classical statistical measures for all pairs of factors including sex and age. The 'FULL' and 'NAF' notations represents the dataset used for computation. 'FULL' dataset is the complete disease data while the filtered 'NAF' dataset contains only those co-morbid occurrences if diseases arising before the onset of depression.

Supplementary Dataset S3 Results of logistic regression. The table contains the Beta coefficients and the p-values. For the computation we used R's glm.

Supplementary Dataset S4 The Bayesian direct multimorbidity map results. Beside the direct edges or connections between the different diseases the table contains other types of connections between the disease pairs. A detailed description of these measures can be found in supplementary material.

References

1. Krueger RF, Markon KE. Reinterpreting comorbidity: A model-based approach to understanding and classifying psychopathology. Annu Rev Clin Psychol. 2006;2:111.
2. Middeldorp C, Cath D, Van Dyck R, Boomsma D. The co-morbidity of anxiety and depression in the perspective of genetic epidemiology. A review of twin and family studies. Psychol Med. 2005;35(05):611-24.
3. Team RC. R: A language and environment for statistical computing. R Foundation for Statistical Computing, Vienna, Austria. 2013. ISBN 3-900051-07-0; 2014.
4. Koller D, Friedman N. Probabilistic graphical models: principles and techniques: MIT press; 2009.
5. Pearl J. Probabilistic reasoning in intelligent systems: networks of plausible inference: Morgan Kaufmann; 2014.
6. Pearl J. Models, reasoning and inference. Cambridge: Cambridge University Press; 2000.
7. Meek C, editor Strong completeness and faithfulness in Bayesian networks. Proceedings of the Eleventh conference on Uncertainty in artificial intelligence; 1995: Morgan Kaufmann Publishers Inc.
8. Glymour CN, Cooper GF. Computation, causation, and discovery: Aaai Press; 1999.
9. Cooper GF, Herskovits E. A Bayesian method for the induction of probabilistic networks from data. Machine learning. 1992;9(4):309-47.
10. Madigan D, Andersson SA, Perlman MD, Volinsky CT. Bayesian model averaging and model selection for Markov equivalence classes of acyclic digraphs. Communications in Statistics--Theory and Methods. 1996;25(11):2493-519.
11. Friedman N. Inferring cellular networks using probabilistic graphical models. Science. 2004;303(5659):799-805.
12. Schadt EE, Lamb J, Yang X, Zhu J, Edwards S, GuhaThakurta D, et al. An integrative genomics approach to infer causal associations between gene expression and disease. Nat Genet. 2005;37(7):710-7.
13. Zhu J, Sova P, Xu Q, Dombek KM, Xu EY, Vu H, et al. Stitching together multiple data dimensions reveals interacting metabolomic and transcriptomic networks that modulate cell regulation. PLoS Biol. 2012;10(4):e1001301.
14. Yeung KY, Fraley C, Young WC, Bumgarner R, Raftery AE, editors. Bayesian Model Averaging methods and R package for gene network construction. Big Data Analytic Technology For

Bioinformatics and Health Informatics (KDDBHI), workshop at the 20th ACM SIGKDD Conference on Knowledge Discovery and Data Mining (KDD); 2014.
15. Yeung KY, Bumgarner RE, Raftery AE. Bayesian model averaging: development of an improved multi-class, gene selection and classification tool for microarray data. Bioinformatics. 2005;21(10):2394-402.
16. Verzilli CJ, Stallard N, Whittaker JC. Bayesian graphical models for genomewide association studies. The american journal of human genetics. 2006;79(1):100-12.
17. Antal P, Millinghoffer A, Hullám G, Szalai Cs FA, editors. A Bayesian view of challenges in feature selection: multilevel analysis, feature aggregation, multiple targets, redundancy and interaction. JMLR Workshop Conf Proc; 2008.
18. Antal P, Millinghoffer A, Hullam G, Hajos G, Sarkozy P, Szalai C, et al. Bayesian, systems-based, multilevel analysis of biomarkers of complex phenotypes: from interpretation to decisions. In: Sinoquet C, Mourad R, editors. Probabilistic Graphical Models for Genetics, Genomics, and Postgenomics: OUP Oxford; 2014. p. 318-62.
19. Giudici P, Castelo R. Improving Markov chain Monte Carlo model search for data mining. Machine learning. 2003;50(1-2):127-58.
20. Gelman A, Carlin JB, Stern HS, Rubin DB. Bayesian data analysis: Taylor & Francis; 2014.
21. Ramos EM, Hoffman D, Junkins HA, Maglott D, Phan L, Sherry ST, et al. Phenotype–Genotype Integrator (PheGenI): synthesizing genome-wide association study (GWAS) data with existing genomic resources. European Journal of Human Genetics. 2014;22(1):144-7.
22. Pinero J, Queralt-Rosinach N, Bravo A, Deu-Pons J, Bauer-Mehren A, Baron M, et al. DisGeNET: a discovery platform for the dynamical exploration of human diseases and their genes. Database-the Journal of Biological Databases and Curation. 2015. doi: 10.1093/database/bav028. PubMed PMID: WOS:000361048600001.
23. Menche J, Sharma A, Kitsak M, Ghiassian SD, Vidal M, Loscalzo J, et al. Uncovering disease-disease relationships through the incomplete interactome. Science. 2015;347(6224):1257601.
24. Liu C-C, Tseng Y-T, Li W, Wu C-Y, Mayzus I, Rzhetsky A, et al. DiseaseConnect: a comprehensive web server for mechanism-based disease–disease connections. Nucleic Acids Res. 2014;42(W1):W137-W46.
25. Smith DJ, Nicholl BI, Cullen B, Martin D, Ul-Haq Z, Evans J, et al. Prevalence and characteristics of probable major depression and bipolar disorder within UK biobank: cross-sectional study of 172,751 participants. PLoS One. 2013;8(11):e75362.
