# Supplementary figures and images for: Comorbidities in the diseasome are more apparent than real: What Bayesian filtering reveals about the comorbidities of depression
Source: PLoS Comput Biol. 2017 Jun 23;13(6):e1005487. doi: 10.1371/journal.pcbi.1005487 (PMC5507322; doi:10.1371/journal.pcbi.1005487)

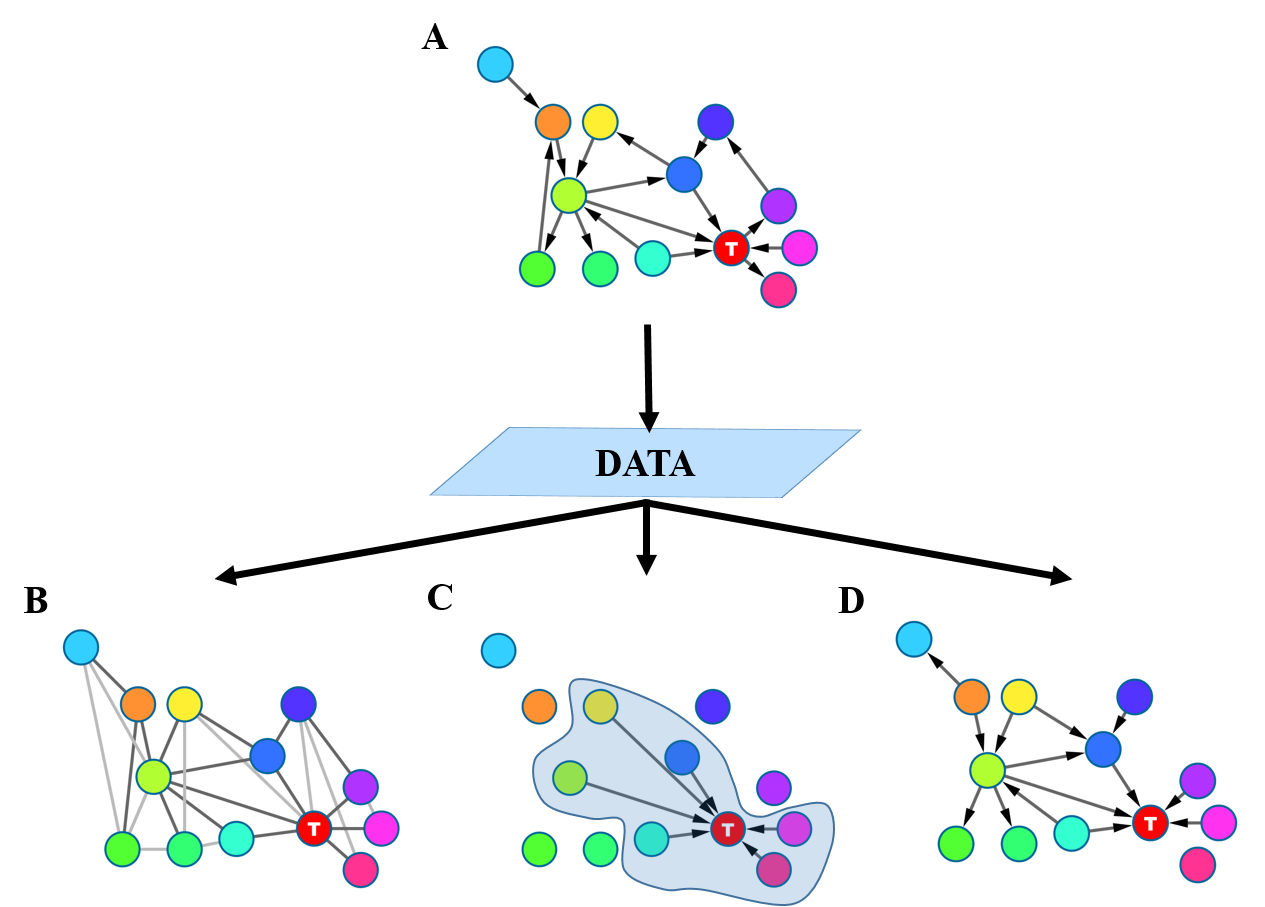

Supplement: S1 Fig — (TIF) [file pcbi.1005487.s002.tif]

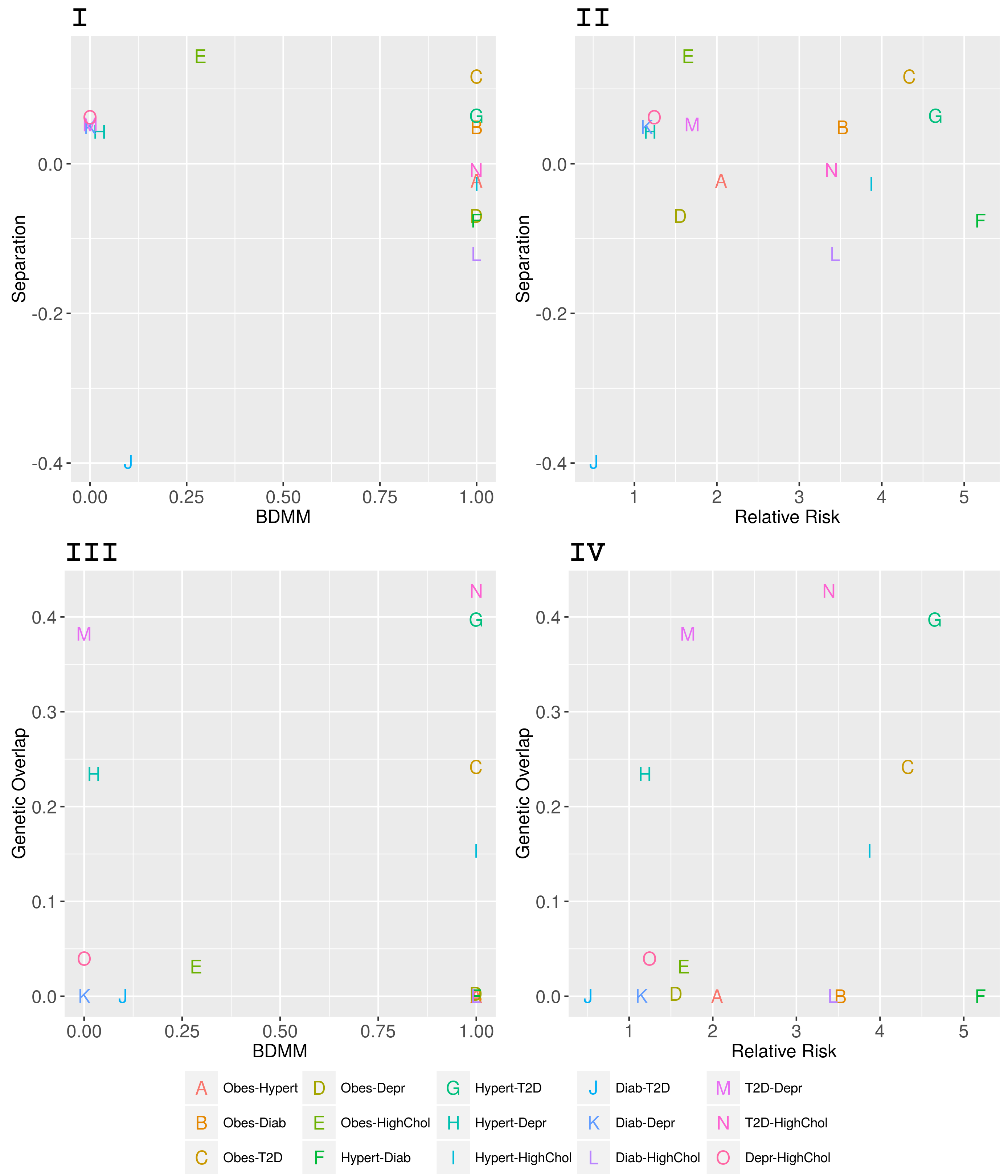

Supplement: S2 Fig — (TIF) [file pcbi.1005487.s003.tif]

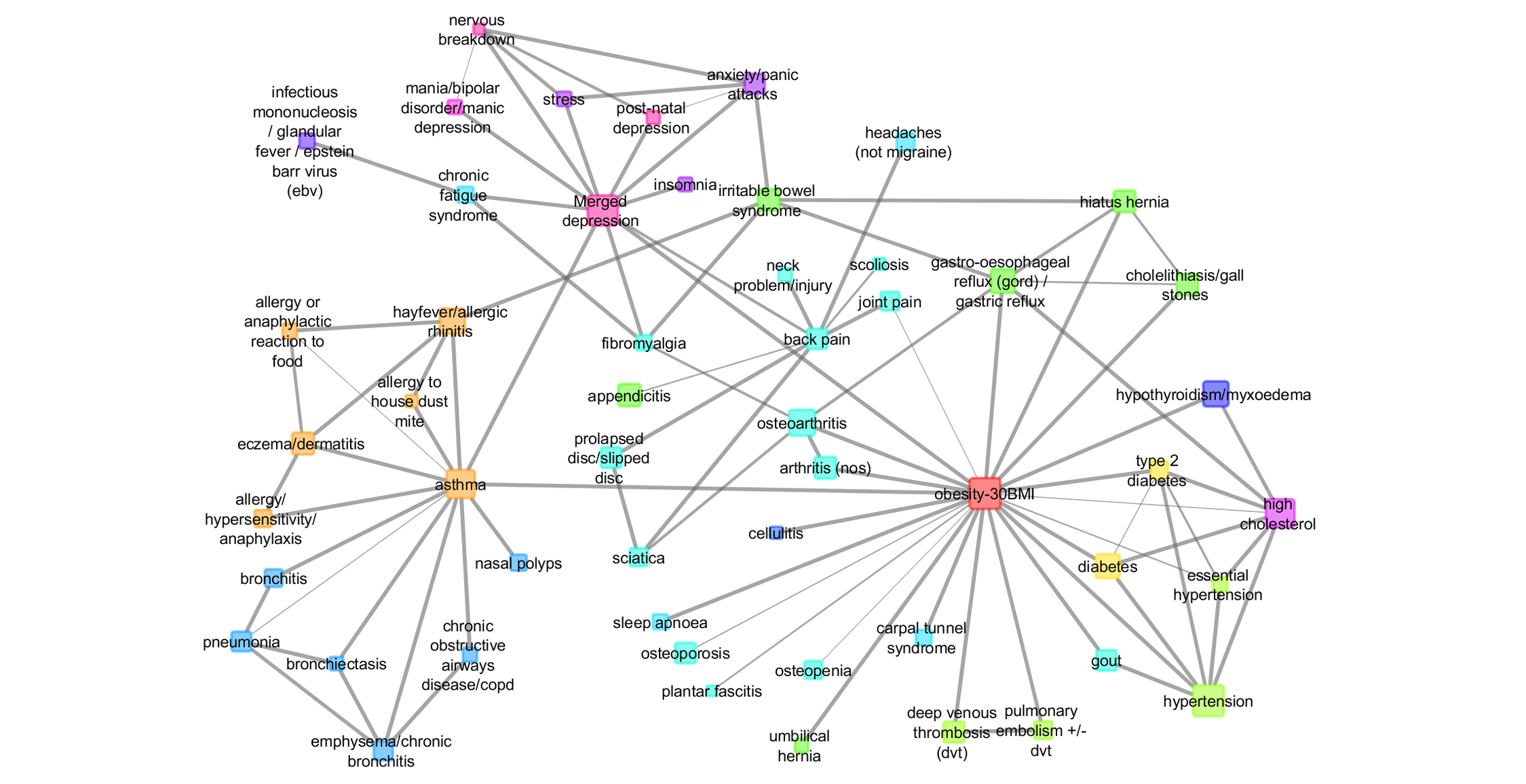

Supplement: S3 Fig — (TIF) [file pcbi.1005487.s004.tif]

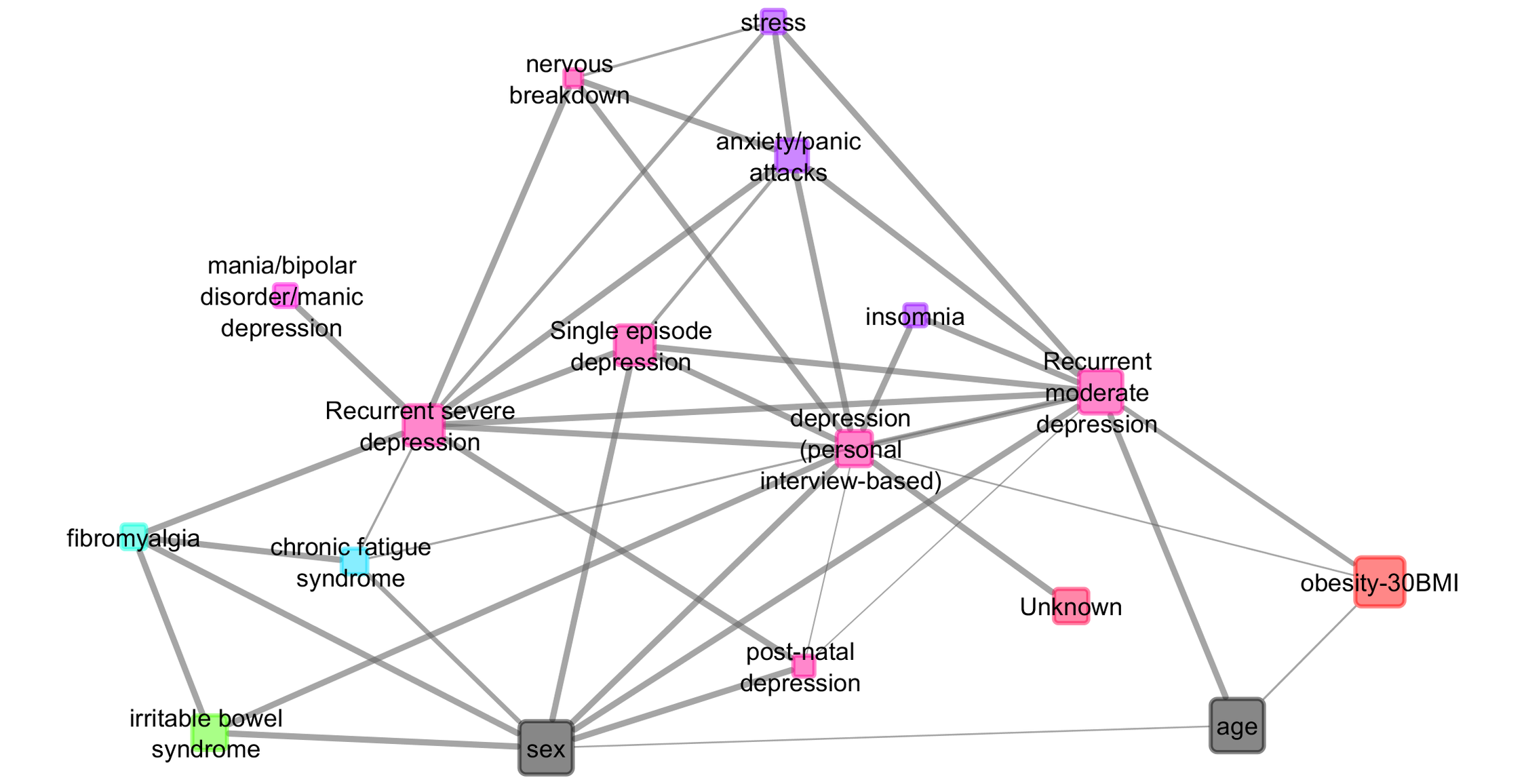

Supplement: S4 Fig — (TIF) [file pcbi.1005487.s005.tif]

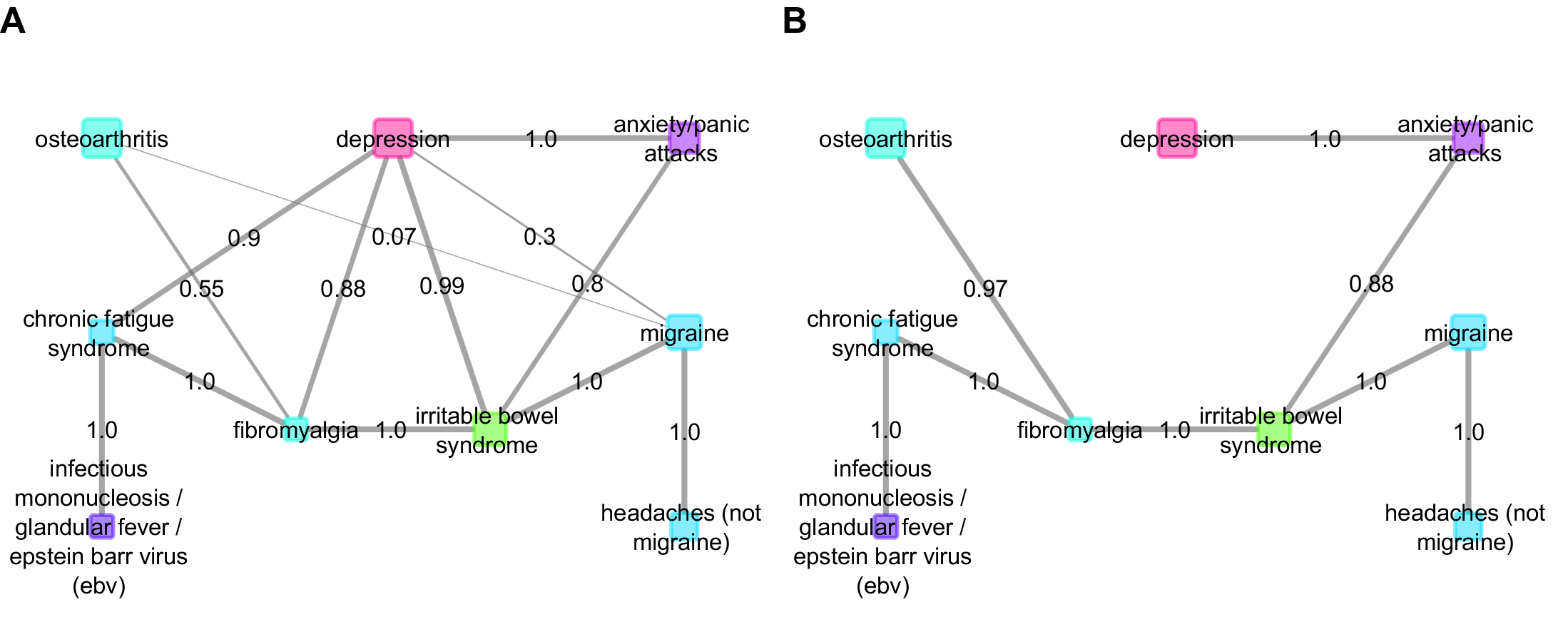

Supplement: S5 Fig — (TIF) [file pcbi.1005487.s006.tif]
